# Behaviour-Based Temperature-Sensor Role Inference and Lagged Weight-Gain Event Prediction in Multi-Year Smart-Hive Telemetry

**DOI:** 10.3390/s26216684

**Published:** 2026-10-22

**Authors:** Sebastian Gorecki, Sławomir Hausman, Piotr Korbel

**Affiliations:** Institute of Electronics, Lodz University of Technology, 90-924 Lodz, Poland; slawomir.hausman@p.lodz.pl (S.H.); piotr.korbel@p.lodz.pl (P.K.)

**Keywords:** precision beekeeping, smart beehive, temperature-sensor role inference, hive mass, thermal buffering, IoT telemetry, operational mass-increase event, lagged prediction, grouped validation, sensor semantics

## Abstract

Long-term smart-hive monitoring is complicated by uncertain temperature-sensor placement and the composite nature of hive-mass changes. We analysed BeeHUB telemetry from 2022–2025 to infer temperature-channel roles and predict daytime mass-increase events (≥0.3 kg). Role inference used fixed heuristics based on thermal offset, variability and ambient correlation. The biological analysis included 35,004 stream-days from 110 telemetry streams; prediction used 20,034 target-complete, weather-matched stream-days. During calendar-defined May–June, warm–stable days had a median inferred internal temperature of 34.8 °C and thermal buffer of 17.0 °C. Among warm–stable days with observed mass change, median daytime increase was 0.35 kg and event frequency was 52.5%. Whole-stream bootstrap intervals supported higher warm–stable mass change and event frequency than four comparison states, but not the sparse warm–unstable state. Across 50 stream-grouped holdouts, a strictly lagged unweighted histogram-gradient-boosting model achieved areas under the receiver operating characteristic curve of 0.809±0.018 in May–June and 0.797±0.026 in summer (mean ± standard deviation), compared with 0.751±0.018 and 0.727±0.030 for previous-day persistence. These findings support conditional thermal-state interpretation and autoregressive prediction, not verified nectar intake or causal thermal effects. Sensor roles lacked placement ground truth, and generalisation to unseen biological colonies has not been established.

## 1. Introduction

Precision beekeeping uses continuous non-invasive measurements to complement manual colony inspections and to support timely management decisions [1,2,3,4]. Current systems may combine hive weight, internal temperature and humidity, acoustics, entrance traffic, and weather. This multimodal setting is attractive, but it also creates a semantic problem: a column name is not necessarily a stable biological measurement. Temperature depends strongly on sensor placement relative to the brood nest, hive walls, and ambient air, and the same nominal channel may behave differently after installation changes or across devices. Placement is therefore part of the measurement model, not a minor implementation detail [5,6].

Early electronic beehive-monitoring systems employed dedicated temperature networks and wireless sensor architectures [7,8,9,10]. More recent platforms integrate multiple sensing modalities, cloud connectivity, and remote data access [11,12,13].

For battery-powered AIoT deployments, however, sensing, local computation and wireless communication must be considered jointly, because the energy cost of transmitting high-volume raw data can outweigh that of local feature extraction [14].

Earlier BeeHUB-related work combined ecological hive insulation with remote monitoring for early anomaly detection [15]. Subsequent studies extended this platform toward large-scale IoT telemetry, edge-aware forecasting and diagnostic analysis [16], as well as multimodal hive-weight forecasting using environmental and acoustic telemetry [17]. Ecological hive insulation has also been evaluated experimentally using IoT-based field telemetry [18]. The present study extends this line of research by explicitly addressing temperature-channel semantic heterogeneity, behaviour-based sensor-role inference, proxy-label limitations and grouped evaluation on held-out telemetry streams.

Honeybee brood thermoregulation is a collective process that produces spatially heterogeneous and location-dependent temperature patterns [19,20,21,22]. Consequently, single-point temperature monitoring can be informative, but its interpretation depends strongly on probe placement, brood distribution, and colony context [23,24,25].

Hive-weight dynamics have a similarly composite interpretation. Within-day change reflects departures and returns of foragers, delivered nectar, pollen and water, evaporation, respiration and moisture exchange. Longer disturbances may also reflect feeding, harvesting, equipment changes, robbing, swarming or scale artefacts [26,27,28]. Consequently, a positive daily change is operationally useful but is not equivalent to a direct observation of floral nectar flow. Mechanistic and statistical models can extract informative structure from weight trajectories [29,30,31,32], while recent multimodal approaches have combined hive-weight history with environmental and acoustic IoT telemetry for short-term forecasting [17]. However, evaluation must respect time order and avoid using target-derived information from the same day.

Hive acoustics and vibrations have been investigated for swarming and colony-state monitoring, although comparability depends on microphone placement, recording hardware and feature-extraction procedures [33,34,35]. More recent embedded approaches demonstrate that spectral descriptors can be extracted directly on resource-constrained sensing nodes and transmitted as compact telemetry rather than continuous raw audio [36].

Honeybee flight and foraging activity are strongly conditioned by temperature, solar radiation and other local weather variables [37,38].

More generally, a sensor observation is interpretable only together with the observed property, sensing procedure and deployment context [39,40].

Prior work has demonstrated complete sensor platforms [41,42], combined sensor–weather monitoring [43,44], and relationships among weight, weather, landscape and traffic [45,46]. Two gaps remain important for heterogeneous commercial deployments. First, most analyses presume that raw temperature-channel names retain a common biological meaning. Second, biological interpretation and operational forecasting are often discussed as though the most predictive feature were also an independent biological explanation. This is especially risky when the target and its strongest predictors are derived from the same physical signal.

Generalisation across deployments is another central issue. Smart-hive models may exploit persistent stream identity, device configuration or site-specific conditions rather than transferable biological signals. Evidence from acoustic monitoring shows that high within-dataset accuracy may coexist with poor extrapolation to unseen hives [47]. Grouping repeated observations by the available deployment identifier is therefore a minimum requirement. In the present archive, however, that identifier denotes a device/telemetry stream rather than a technician-verified longitudinal colony; the evaluation therefore supports generalisation to held-out streams, not a definitive claim about unseen biological colonies.

The present work is complementary to, but methodologically distinct from, our previous studies of the BeeHUB platform. Earlier work addressed large-scale telemetry architecture, forecasting and diagnostics [16], multimodal hive-weight forecasting [17], communication-efficient embedded acoustic processing [36], and field evaluation of hive thermal insulation [18]. In contrast, the present study focuses on semantic heterogeneity of temperature channels across long-term field deployments, behaviour-based sensor-role inference, thermal-state phenotyping, and strictly lagged evaluation on held-out telemetry streams.

This study evaluates a multi-year BeeHUB archive using a two-layer framework. The *biological-context layer* interprets temperature-channel behaviour and thermal buffering without assuming fixed channel semantics. The *operational-prediction layer* evaluates whether previous-day and previous-week history can discriminate a next-day operational daytime mass-increase event. The endpoint measures short-term mass increase; no direct nectar intake, floral phenology or inspection label was available as ground truth.

The study addresses three questions:

1. Can per-stream temperature-channel behaviour distinguish ambient-like, internal-like and colony-core-like signals in a heterogeneous field archive, and how uncertain are these assignments?
2. Is a warm and thermally stable inferred state associated with stronger mass-increase indicators during the calendar-defined May–June period?
3. How does discrimination of a next-day operational event differ among lagged weather history, lagged non-weight BeeHUB signals and lagged weight history under repeated evaluation on held-out telemetry streams, and how much does the full model improve on persistence?

The main contribution is a deployment-level method for resolving semantic heterogeneity in temperature sensor channels, coupled with an evaluation framework that separates inferred sensor semantics, descriptive biological context and autoregressive operational prediction. Specifically, the study (i) infers sensor roles from per-stream thermal behaviour rather than database labels, (ii) quantifies assignment uncertainty and selection effects, (iii) derives season-aware telemetry phenotypes, and (iv) compares contextual and autoregressive predictors using repeated grouped holdouts, whole-stream uncertainty estimates and a persistence baseline.

## 2. Materials and Methods

### 2.1. Study Design and Analysis Provenance

The study is a retrospective observational analysis of routinely collected BeeHUB telemetry. The analytical notebook, its embedded outputs and the frozen intermediate tables were audited together; all thresholds and model settings reported below are taken from executable code rather than inferred from figures. Direct inspection labels were not collected. The analysis is therefore archive-level evidence and the sensor-role labels are behavioural inferences rather than externally validated positions.

**Figure 1:**
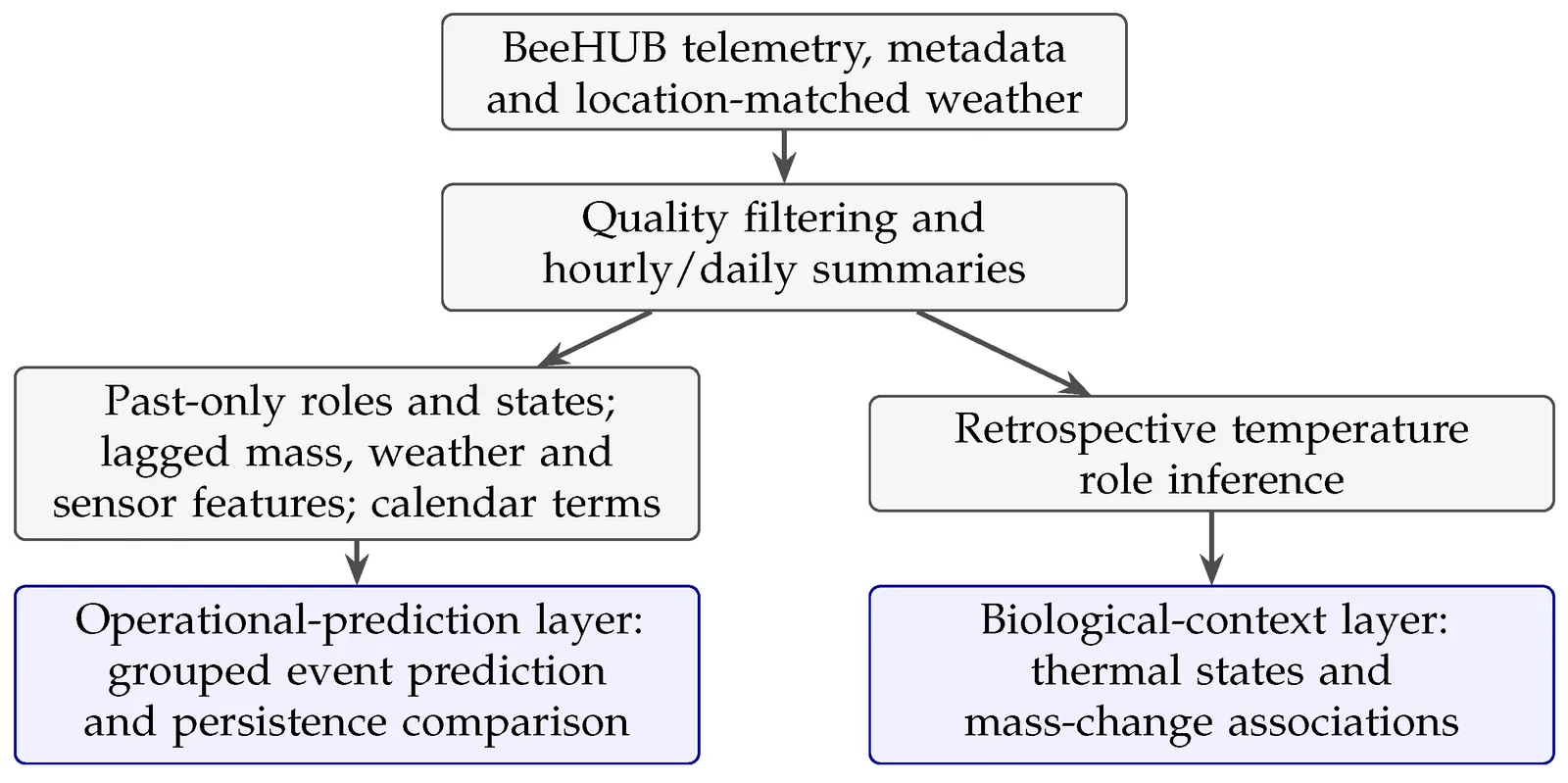
Analysis framework separating descriptive biological context from operational prediction. The biological branch uses retrospective temperature-role assignments. The predictive branch uses lagged mass, weather and sensor features, including lagged thermal states constructed with past-only role mappings, together with calendar terms. Predictive feature availability is checked against the midnight forecast origin. The modelling cohort remains retrospectively selected. Neither inferred thermal states nor predictive performance establishes a causal mechanism or direct nectar intake. Grey boxes denote data-processing and feature- or role-construction steps, whereas blue boxes denote the two analytical outcome layers.

The analysis was organised into four stages: (i) daily aggregation and weather matching; (ii) behaviour-based interpretation of temperature channels; (iii) descriptive thermal-state analysis; and (iv) grouped predictive diagnostics. Figure 1 shows the separation between biological context and operational prediction.

**Figure 2:**
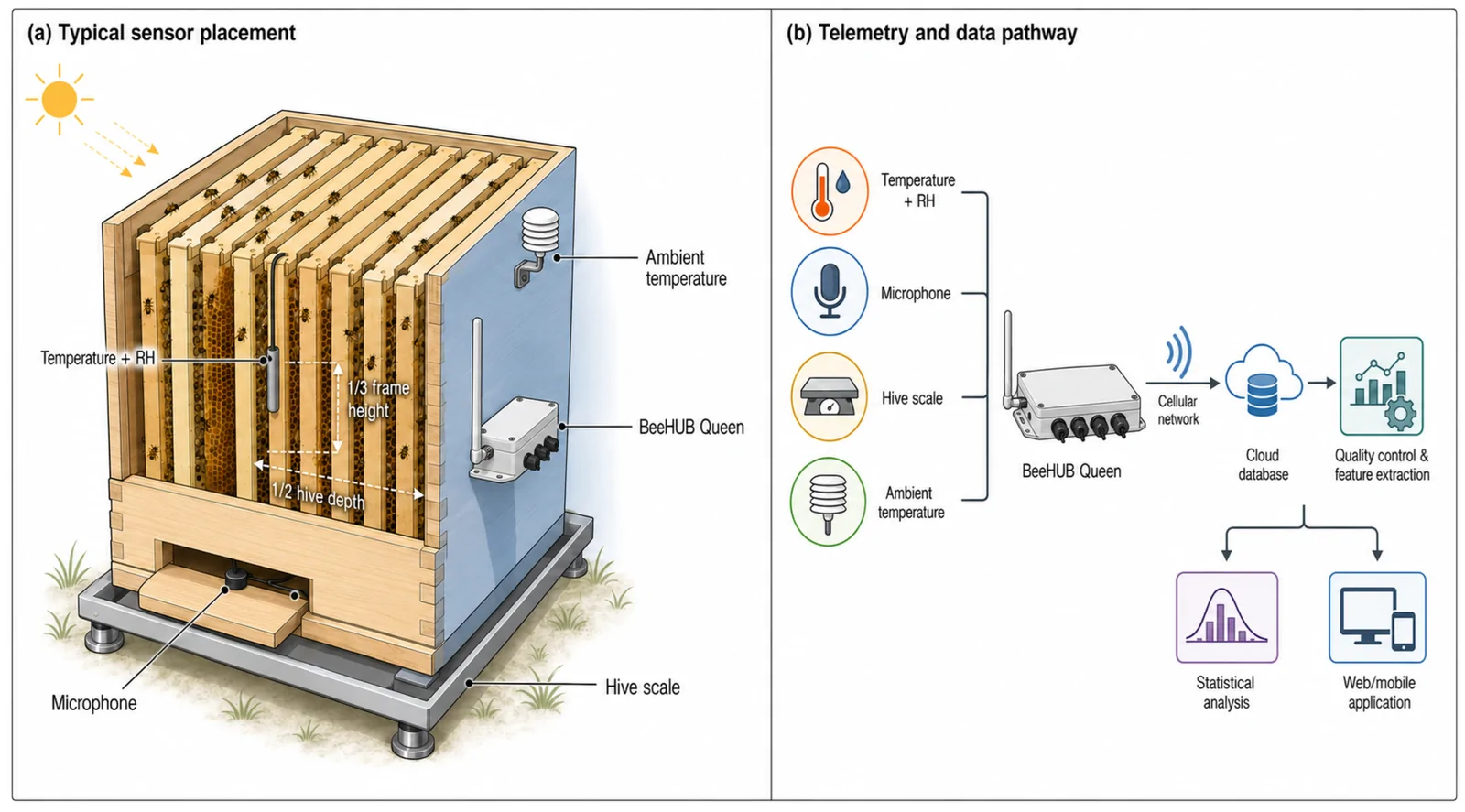
Smart-hive sensing layout and telemetry pathway. (**a**) Recommended installation: the temperature–relative-humidity probe is placed between the central brood frames, approximately one-third of the frame height below the top bars and midway along the hive depth. The microphone is installed near the entrance, the load-cell scale beneath the hive, and the ambient-temperature probe and BeeHUB Queen on a shaded external wall. (**b**) BeeHUB Queen transmits measurements via a cellular network to a cloud database for quality control, feature extraction and analysis. Historical sensor placements may have differed.

Figure 2 summarizes the recommended field installation. The combined temperature–relative-humidity probe was positioned within the central brood-frame region, approximately one-third of the frame height below the top bars and halfway between the entrance and rear hive walls. The microphone was installed inside the lower hive body adjacent to the entrance, whereas the load-cell platform supported the complete hive. The ambient-temperature probe and the BeeHUB Queen telemetry unit were mounted on the shaded external wall, with the telemetry unit positioned at least 25 cm above ground level, in accordance with the manufacturer’s installation guidance [48]. Because installation histories were not independently verified for every device, this diagram describes the recommended configuration and should not be interpreted as ground-truth placement for every archived temperature channel.

### 2.2. Telemetry Archive and Unit of Analysis

The biological and predictive analytical tables covered 4 January 2022 to 2 December 2025. Archive-discovery, unit-eligibility and retrospective channel-role audits describe the broader source export; their file and identifier counts are not counts restricted to that analytical interval. Selection comparisons and modality-exposure summaries in this revision were restricted to the stated analytical interval. Available telemetry included hive mass, ambient and nominal internal temperature, internal relative humidity, and aggregated acoustic frequency and amplitude indicators. Sampling frequency varied among devices. Raw timestamps were parsed as UTC, converted to Europe/Warsaw local time with daylight-saving transitions, and aggregated to hourly medians. Overlapping exports were prioritised by source and deduplicated by the archive field hive_id and local hourly timestamp.

The field hive_id represents an operational device/telemetry stream, not a technician-verified persistent biological colony. Source files belonging to the same identifier were consolidated before deduplication. The archive did not retain longitudinal device–colony assignment histories, so a device relocation between colonies or replacement of a device within one colony could not be reconstructed. The grouping unit was therefore the telemetry-stream identifier and the daily analytical unit was the stream-day. Holding out a stream prevents that recorded identifier from occurring in both training and test data, but it cannot guarantee that the underlying biological colony was never represented under another identifier.

The broad discovery scan identified 1286 files. Of the 45 files not standardised as telemetry inputs, 40 were empty or near-empty telemetry files and contributed zero loaded records and zero stream-days, whereas five were cached Open-Meteo responses outside the telemetry-import scope. The remaining 1241 telemetry files were successfully loaded. They formed 1032 standardised stream groups: 1031 nonblank identifiers and one blank-identifier group. The blank group contributed two channel-role evaluations and 328 provisional active-season daily records and was removed before all revised biological and predictive summaries.

After this exclusion, the role-corrected biological table contained 35,004 active-season stream-days from 110 nonblank streams. Of these, 27,756 stream-days (79.3%) were matched to weather. The strict temporal modelling subset contained 20,034 target-complete, weather-matched stream-days. These denominators are not interchangeable. Table 1 reports seasonal sizes, Table 2 summarises the retained sensing modalities, and Table 3 gives the complete analytical flow.

**Figure 3:**
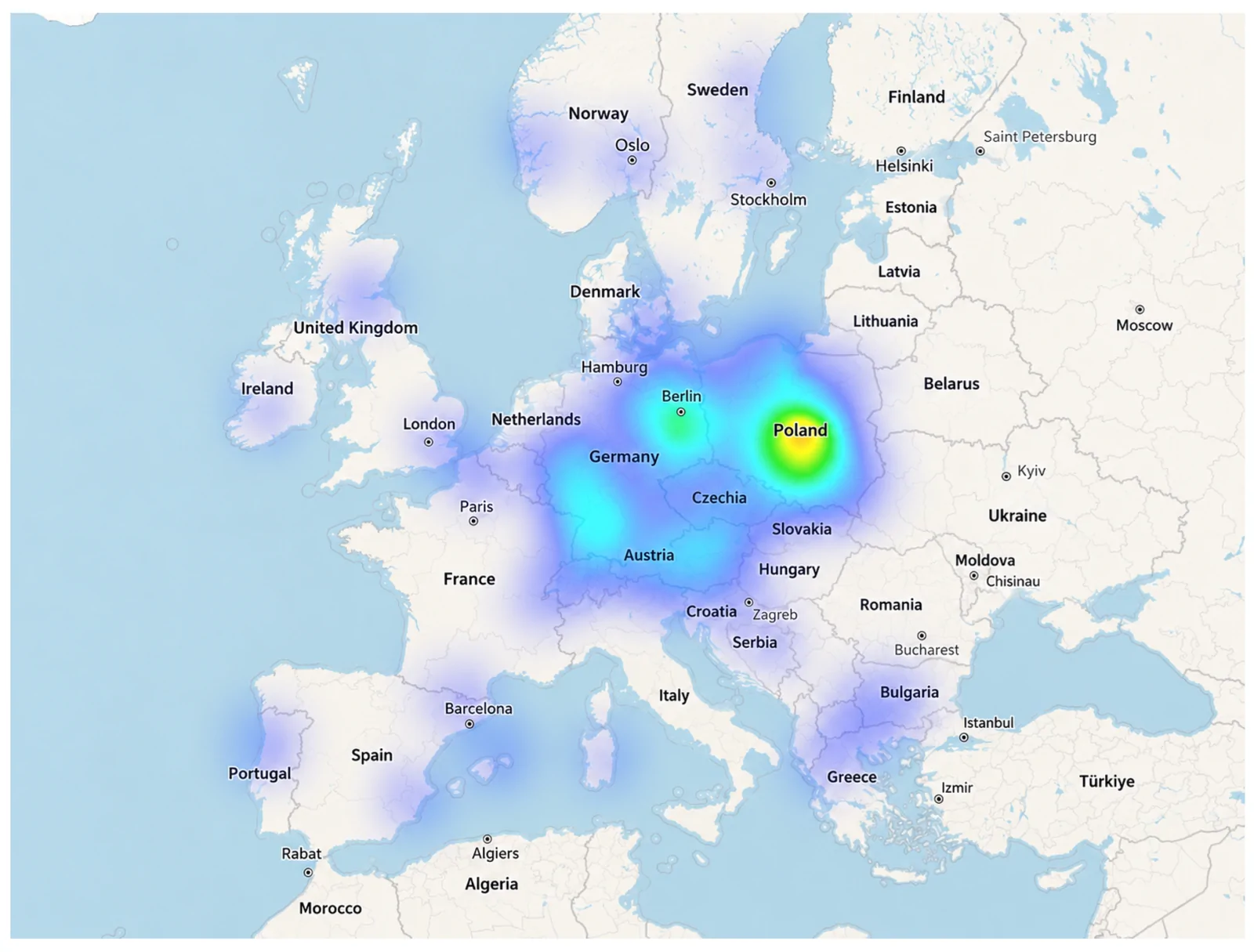
Anonymised coverage of the weather-linked modelling subset. The 80 rounded coordinate pairs spanned 42.08–54.58° N and 0.49–24.74° E and are displayed as a density surface rather than individual sites. Warmer colours indicate a higher density of rounded coordinate groups, whereas cooler colours indicate lower density. Rounded coordinates are approximate location groups, not verified apiary identifiers.

Within the weather-linked modelling subset, 87 stream assignments were associated with 80 rounded coordinate pairs spanning 42.08–54.58° N and 0.49–24.74° E. Country and altitude metadata were not sufficiently complete for country-level or altitude-stratified analysis. Coordinates were rounded for privacy and were used only as approximate location groups; they must not be interpreted as verified apiary identifiers or as a probability sample of European apiaries (Figure 3).

Table 2 summarises the retained sensing modalities and their metrological interpretation.

The complete archive, identifier and mass-unit audit is provided in Supplementary Table S1.

### 2.3. BeeHUB Microclimate and Hive-Mass Sensing

**Figure 4:**
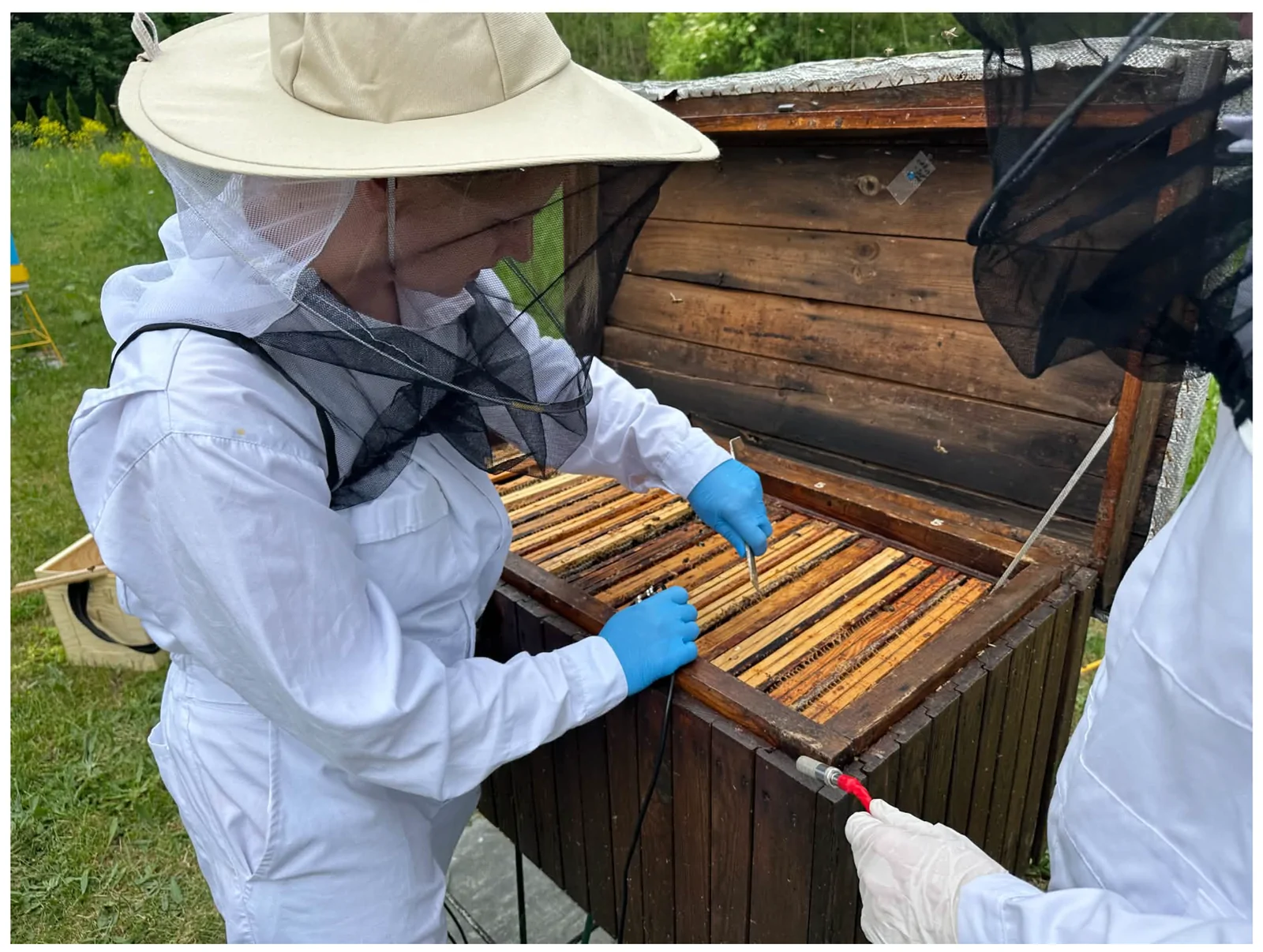
Field installation of wired BeeHUB sensor probes in an occupied hive. The photograph illustrates the practical placement variability that motivated behaviour-based interpretation of temperature channels. Photograph: Intelligent Hives Sp. z o.o.

Dedicated temperature measurements were acquired with DS18B20 digital thermometers (Maxim Integrated Products, Inc., San Jose, CA, USA) communicating over a 1-Wire bus. The component supports measurements from −55 to +125 °C, with a manufacturer-specified accuracy of ±0.5 °C from −10 to +85 °C. Its programmable 9–12-bit conversion corresponds to increments from 0.5 °C to 0.0625 °C, respectively [49]. Probes could serve ambient or within-hive roles depending on installation. Because the archive did not retain a technician-verified mapping between every physical probe and database field, these component specifications do not remove the channel-semantic problem addressed in this study (Figure 4).

Internal relative humidity was measured with the Sensirion SHT20 digital humidity sensor (Sensirion AG, Stäfa, Switzerland) using an I2C interface. At its default 12-bit humidity setting, the nominal resolution is 0.04% RH; the typical accuracy is ±3.0% RH at 25 °C and 3.0 V, excluding hysteresis and long-term drift. The extended measurement range is 0–100% RH, but the manufacturer defines 0–80% RH as the normal range and reports that prolonged exposure above 80% RH can produce a reversible offset. The typical long-term drift is below 0.25% RH per year under normal operating conditions [50]. Within the analytical interval, 25.2% of 200,195 days with a valid internal-humidity daily maximum had that maximum above 80% RH. No device-specific drift correction was available. Accordingly, extreme within-hive humidity values were treated as contextual telemetry rather than laboratory-reference measurements.

Hive mass was measured with a custom 45 cm × 45 cm instrumented frame incorporating miniature strain-gauge load cells rated at 50 kg per sensing element. The retained supplier specification lists dimensions of 28 mm × 28 mm × 8 mm, output sensitivity of 1.1±0.15 mV/V, comprehensive error of 0.2% FS, repeatability of 0.1% FS, hysteresis and nonlinearity of 0.2% FS, creep of 0.1% FS over 3 min, an excitation range of 5–10 V, an operating range of −10 to +50 °C, and an overload rating of 150% FS. These are component-level ratings, not the verified accuracy of the assembled hive scale. The latter also depends on frame mechanics, load distribution, analogue acquisition, calibration and temperature compensation. The analytical archive contained mass values in kilograms but did not retain per-device calibration certificates, reference-load tests or maintenance histories.

### 2.4. BeeHUB Acoustic Sensing Subsystem

The platform-level BeeHUB acoustic-processing framework has been described separately [36]. That work describes an ESP32-class sensing node with an SPH0645LM4H digital MEMS microphone and an I^2^S interface. Depending on the monitoring objective and installation constraints, the microphone may be positioned near the hive entrance or inside the hive. These platform-level descriptions do not establish the microphone revision, mounting configuration or acquisition settings associated with every record in the present archive.

The previously reported signal-processing configuration used an analysis sampling frequency of 8192 Hz and 4096-sample FFT buffers [36]. These parameters correspond to a 0.5-s window, 2-Hz FFT-bin spacing and a Nyquist frequency of 4096 Hz. The configuration used a Hann window and real-valued FFT, with extraction of dominant frequency, peak and mean spectral amplitude, spectral centroid, spectral entropy and band energy; the earlier colony-activity analysis focused on the 200–400 Hz band.

These processing parameters must not be interpreted as a verified microphone-acquisition specification for the complete 2022–2025 archive. The frozen export did not retain the per-record acquisition and firmware metadata needed to reconstruct the hardware sampling configuration or establish whether sample-rate conversion had been applied. The present study analysed archived acoustic descriptors rather than raw waveforms or complete spectra. After quality control, the retained dominant-frequency descriptor was restricted to 20–1200 Hz.

Only compact descriptors are transmitted through LTE-M or NB-IoT rather than continuous raw audio [36]. This design is consistent with the broader cross-layer principle that edge computation should be evaluated jointly with communication energy and payload reduction in constrained AIoT sensing systems [14].

These specifications explain the platform architecture and the feature-level character of the frequency and amplitude fields available to the present analysis. They do not establish that every 2022–2025 record originated from the same microphone revision, mounting position, firmware version or FFT configuration: the frozen export did not preserve those identifiers. Moreover, amplitude was retained in relative device units and was not calibrated to sound-pressure level. No device-specific amplitude normalisation, physical calibration or longitudinal drift correction was available. Training-fold imputation and standardisation used in predictive models harmonised numerical scale but must not be interpreted as physical calibration. The acoustic variables were consequently used as contextual, lagged predictors and were not assigned a direct biological label.

Additional acoustic-amplitude and relative-humidity exposure diagnostics are reported in Supplementary Table S10.

### 2.5. Data Preparation, Quality Control and Seasons

Device eligibility required at least 14 observed days. Weight analysis additionally required at least 72 valid weight hours, at least 5% non-missing weight coverage and a within-stream 95th–5th percentile mass range of at least 0.20 kg. Identifiers or source filenames containing test, demo, dummy, bench, lab, additional_temperature, temperature_data, weather, soil or tracker were flagged as possible non-hive records and excluded where configured. Per-stream weight units were inferred from the raw median. Inclusive medians from 5 to 250 were treated as kilograms (723 streams, of which 692 subsequently met all weight-analysis criteria), whereas medians from 5000 to 250,000 were divided by 1000 (52 streams, of which 44 were retained). Streams with medians below 5 (178 streams), in the unresolved interval above 250 and below 5000 (six streams), or above 250,000 (zero streams) were excluded from weight-derived analyses; 73 additional streams had no usable weight median. Thus, 736 streams entered weight analysis after unit interpretation and the remaining eligibility criteria.

The broad technical acceptance ranges were 5–250 kg for mass, −40 to 80 °C for nominal internal temperature, −40 to 60 °C for ambient temperature, 0–100% for relative humidity, 0–2000 Hz for acoustic frequency and 0–5000 arbitrary units for acoustic amplitude. For the final biological table, inferred internal daily means were restricted to 5–45 °C, minima to 0–45 °C, maxima to 5–50 °C and daily standard deviations to 0–15 °C; ambient values were restricted to −35 to 50 °C. Humidity was restricted to 1–100%, acoustic frequency to 20–1200 Hz and amplitude to 1–5000 arbitrary units. Values between −0.5 and 0.5 in internal temperature or acoustic channels were treated as missing-device artefacts. Daytime gain was restricted to −5 to 10 kg and 24 h and night-time changes to −10 to 10 kg. Missing values outside these rules were set to missing; they were not interpolated for descriptive analyses. Model-specific imputation is described below.

Seasons were fixed from local calendar month before outcome analysis: winter (December–February), early spring (March–April), the calendar-defined May–June period (stored as main_flow), summer (July–August), autumn (September–October) and late autumn (November). The four active-season bins were analysed in this paper. These are reproducible calendar strata for the observed geographic subset, not site-specific phenological labels. In particular, the May–June label must not be interpreted as a validated onset and end date of the principal forage period throughout temperate Europe.

### 2.6. Weather Enrichment

Historical daily weather was retrieved by rounded coordinate and date from the Open-Meteo archive interface [51]. Variables included mean, minimum and maximum air temperature; apparent temperature; precipitation and rain sums; mean wind speed and maximum gust; shortwave radiation; evapotranspiration; mean relative humidity; dew point; cloud cover; and vapour pressure deficit. Retrieved responses were cached and joined to daily hive records by date and rounded coordinates.

### 2.7. Weight-Derived Variables and Operational Event Label

For telemetry stream *s* on date *d*, daytime mass change was defined in Equation (1) as

(1) $$\Delta W_{s,d}^{\mathrm{day}}=W_{s,d}^{\mathrm{evening}}-W_{s,d}^{\mathrm{morning}},$$

The hourly mass series was first smoothed within each stream using a centred rolling median over three retained hourly rows, with at least one non-missing value required per window. The morning value was the median from 05:00–08:00 local time (inclusive), the midday value from 11:00–14:00 and the evening value from 18:00–21:00.

Daily mass coverage was the proportion of retained hourly rows on that date with a non-missing smoothed mass value. Hours absent from the retained index were not included in the denominator. Coverage was calculated after smoothing; consequently, a row with missing original mass could contribute a non-missing smoothed value. The coverage calculation used the smoothed mass series, not the event-masked hourly-rate series. A day was flagged as weight-valid when this proportion was at least 50%, no more than two primary large-jump flags occurred, and both morning and evening summaries were available.

Hours with positive and negative mass rate were counted from 08:00–19:00 when the cleaned hourly rate was greater than +0.05 or less than −0.05 kg h^−1^, respectively.

The hourly rate was the change in smoothed mass divided by the actual elapsed time. An absolute rate above 3.0 kg h^−1^ was treated as a technical or management event; that hour and its immediate predecessor and successor were removed from rate-based summaries. The archive did not contain independent feeding, harvesting or hive-opening labels. Consequently, this rule suppresses abrupt interventions but cannot identify all gradual feeding, multi-hour harvests, equipment changes or sensor drift. Net 24 h change was next-morning minus current-morning mass, and night change was next-morning minus current-evening mass.

The binary target was defined in Equation (2) as

(2) $$Y_{s,d}=\left\{\begin{array}{cc} 1, & \Delta W_{s,d}^{\mathrm{day}}\geq 0.3\;\mathrm{kg},\\ 0, & \Delta W_{s,d}^{\mathrm{day}}<0.3\;\mathrm{kg},\\ \mathrm{missing}, & \Delta W_{s,d}^{\mathrm{day}}\;\mathrm{not}\;\mathrm{observed}. \end{array}\right.$$

The 0.3 kg cutoff was specified in the operational pipeline as a pragmatic alert threshold; it was not estimated from the present outcomes and does not represent a known biological boundary. The endpoint measures only short-term daytime mass increase and was not equated with observed nectar intake or verified foraging input. Sensitivity analyses repeated biological event-rate contrasts and predictive evaluation for thresholds from 0.10 to 1.00 kg in 0.10 kg increments. Days with missing daytime mass change were excluded before every event definition.

### 2.8. Behaviour-Based Interpretation of Temperature Channels

Raw temperature-column names were not assumed to encode a fixed sensor position. No technician-verified sensor-position labels were available; all roles were inferred heuristically from thermal behaviour.

The role audit used active-season hourly medians after broad filtering to −30–45 °C for ambient temperature and 0–50 °C for each candidate channel. For each telemetry stream *r*, candidate channel *c* and day *d*, daily means and ranges were calculated. A valid role-audit day required at least eight retained hourly rows with concurrent, non-missing candidate-channel and ambient-temperature values. Daily means and ranges were calculated from all available values of each channel within that eligible day, not only from the concurrent rows used for the eligibility check.

All summaries were computed over the complete available active-season history of a stream–channel pair, and one time-invariant role was assigned to that pair. This stabilises the estimate when daily coverage is sparse but cannot identify sensor repositioning or hardware replacement within the observation period; a changed installation can therefore be represented by a mixture of behaviours. The score components are heuristic similarities, not calibrated posterior probabilities. For each stream–channel pair we then calculated

(3) $$\begin{array}{cccc} N & =\mathrm{number}\;\mathrm{of}\;\mathrm{valid}\;\mathrm{days}, & T & ={\mathrm{median}}_{d}\left({\bar{T}}_{c,d}\right),\\ D & ={\mathrm{median}}_{d}\left({\bar{T}}_{c,d}-{\bar{T}}_{o,d}\right), & R & ={\mathrm{median}}_{d}\left(T_{c,d}^{\max}-T_{c,d}^{\min}\right),\\ R_{o} & ={\mathrm{median}}_{d}\left(T_{o,d}^{\max}-T_{o,d}^{\min}\right), & q & =R/R_{o},\\ \rho & =\rho_{S}\left({\bar{T}}_{c,d},{\bar{T}}_{o,d}\right), & & \end{array}$$

where *o* denotes the ambient channel and Equation (3) summarises the stream–channel behavioural descriptors.

The range ratio *q* was stored as missing when either range was missing or $R_{o}\leq 0$. Spearman correlation was stored as missing when fewer than three paired daily means were available or either daily-mean series was constant.

Missing descriptors did not automatically exclude a stream–channel pair from role assignment. A missing *q* contributed zero to the range-similarity component of $S_{\mathrm{ext}}$. For scoring, $\rho_{c}=0$ when ρ was missing; otherwise, $\rho_{c}=\min\left\{\max\left(\rho ,0\right),1\right\}$. Thus, a missing correlation produced a value of one for the $1-\rho_{c}$ components. The remaining helper functions returned zero for missing inputs. These numerical fallbacks must not be interpreted as measured decorrelation. The minimum-day requirement and ordered role-assignment rules were then applied to the resulting scores.

The following bounded helper functions reproduce the implementation:

(4) $$\begin{array}{cc} Z_{0}\left(x;a\right) & =\max\{0,1-\min(|x|/a,1)\},\\ G(x;a) & =\min\{\max(x/a,0),1\},\\ L(x;a) & =\max\{0,1-\min(x/a,1)\}, \end{array}$$

and P(x;l,u,p) is zero outside [l,u], increases linearly from zero at *l* to one at *p*, and decreases linearly to zero at *u*. Using the missing-value conventions stated above, the three scores in Equation (5) were

(5) $$\begin{array}{cc} S_{\mathrm{ext}}= & 0.45\rho_{c}+0.35Z_{0}\left(D;3.0\right)+0.20Z_{0}\left(q-1;0.7\right),\\ S_{\mathrm{int}}= & 0.30G\left(D;4.0\right)+0.30P\left(T;12,38,30\right)+0.20\left(1-\rho_{c}\right)+0.20P\left(R;0.5,10,3\right),\\ S_{\mathrm{core}}= & 0.35P\left(T;26,37.5,34.5\right)+0.25L\left(R;5.0\right)+0.25G\left(D;7.0\right)+0.15\left(1-\rho_{c}\right). \end{array}$$

The weights, helper-function scales and decision cutoffs were specified as fixed heuristic parameters from bee-biological plausibility and engineering interpretation; no technician-labelled placement subset and no outcome-based optimisation were used. For $S_{\mathrm{ext}}$, ambient correlation received the largest weight (0.45), followed by near-zero median offset (0.35) and similarity of daily range (0.20). For $S_{\mathrm{int}}$, warm offset and a broad plausible internal-temperature range each received 0.30, whereas decorrelation from ambient and moderate daily variability each received 0.20. For $S_{\mathrm{core}}$, brood/core-compatible temperature received 0.35, stability and strong positive offset each received 0.25, and ambient decorrelation received 0.15. The 3, 4 and 7 °C offset scales encode increasingly strong buffering; the internal-temperature support 12–38 °C with a 30 °C peak is deliberately broad, whereas the core support 26–37.5 °C with a 34.5 °C peak reflects brood-area thermal plausibility [19,20,21,22]. The 0.65, 0.55 and 0.45 role cutoffs form a conservative first-match hierarchy, and $S_{\mathrm{int}}\geq 0.60$ was used only as a stronger-evidence threshold for the high-confidence label. Robustness was examined by shifting decision cutoffs by ±0.05, helper scales and piecewise ranges by ±10%, temperature peaks by ±1 °C, varying the high-confidence cutoff by ±0.05, and assigning equal component weights (Supplementary Table S3).

Table 4 gives the exact, ordered decision rule. “Previous rule false” is material because the implementation used first-match precedence rather than independent rectangular thresholds.

For each stream, candidate channels with N≥30 that were not ambient-like were preferred. The internal channel was the candidate with, in order, the greatest $S_{\mathrm{int}}$, greatest $S_{\mathrm{core}}$ and greatest *N*; if all candidates were ambient-like, the same ordering was applied to all candidates. The heuristic mapping-confidence tier was high when the selected role was internal-hive-like or colony-core-like and $S_{\mathrm{int}}\geq 0.60$, low when the selected role was mixed/uncertain or ambient-like, and medium otherwise. Only high- and medium-confidence mappings were used for the role-corrected biological table. These tiers describe rule-based evidence strength and are not statistical confidence levels.

For the core-to-ambient diurnal profile, a second channel was selected independently using the same candidate eligibility and preference for non-ambient-like channels. Candidates were ranked in descending order of $S_{\mathrm{core}}$, then $S_{\mathrm{int}}$, and then *N*. If no non-ambient-like candidate was available, all candidates with N≥30 were considered. This core-score-selected channel was not required to have the assigned role colony-core-like.

Diurnal profiles used streams with high- or medium-confidence internal-channel mappings; no separate core-channel confidence criterion was imposed. The core-to-ambient difference was calculated within each retained hourly row with both measurements available. Observations were pooled by calendar period and hour of day, and the median and 25th–75th percentiles were calculated. Streams were not weighted equally: their contributions depended on the number of available hourly observations.

Role-score separation was summarised by the margin between the largest and second-largest of the three role scores; a margin below 0.10 was flagged as weak separation. For a selected mapping, this statistic was computed from the three scores of the selected channel. Because role assignment follows thresholded first-match rules rather than a maximum-score rule, the top-two score margin need not favour the assigned role. It is not a measure of distance from the final decision boundary. The score margin and mapping-confidence tiers are heuristic descriptors, not posterior probabilities, calibrated confidence measures or statistical confidence intervals. As a separate temporal-stability diagnostic, roles were recomputed independently by year from the retained daily temperature summaries, requiring at least 30 days with paired candidate-channel and ambient daily means within a year. The within-day eight-concurrent-hour criterion of the primary retrospective audit was not reapplied in this daily-summary analysis. Annual disagreement was interpreted as instability of the behaviour-based assignment, not as proof of physical repositioning, because colony state, ambient conditions and coverage can also change across years. To evaluate propagation into the thermal-state analysis, the May–June contrasts were repeated after restriction to high-confidence mappings, selected-score margins of at least 0.10, annually stable multiyear mappings, and mappings that were both high-confidence and annually stable (Supplementary Table S4).

This procedure is described as *behaviour-based role inference*, not role validation. No technician-verified sensor-position audit was available to estimate sensitivity or specificity. A wrong selected role can alter the derived internal temperature, thermal buffer and daily state simultaneously; the resulting state frequencies and mass-change contrasts are therefore conditional on the mapping rule and the selected 110-stream subset.

### 2.9. Inferred Thermal States

Daily internal temperature level, within-day stability and buffering relative to ambient temperature were combined into six substantive descriptive states plus an unknown-data state. For stream *r* on day *d*, thermal buffering was defined in Equation (6) as

(6) $$B_{r,d}={\bar{T}}_{r,d}^{\mathrm{internal}}-{\bar{T}}_{r,d}^{\mathrm{ambient}},$$

where daily temperature summaries were arithmetic means and $s_{r,d}^{\mathrm{internal}}$ below denotes the standard deviation of available hourly medians. Table 5 gives the exact first-match rule used in the final analysis.

The overlap between rules 4 and 5 is intentional in the published hierarchy: a day with $28\leq T_{i}<$ 30 °C, B≥ 5 °C and $s_{i}>$ 5 °C is classified as active internal before the warm-unstable rule is evaluated. As a sensitivity analysis, these two rules were reversed. This changed 94 of 35,004 stream-days (0.27%), including 28 of 8825 May–June days (0.32%), all from active-internal to warm–unstable; all other assignments were unchanged (Supplementary Table S6).

The warm-stable state denotes a high inferred internal temperature, limited within-day variation and positive buffer. It is a telemetry phenotype, not a diagnostic label of colony health, brood amount or queen status.

Associations between thermal state and weight-derived variables were summarised by medians and event proportions. Primary uncertainty intervals used 2000 whole-stream cluster-bootstrap resamples (seed 42). Each draw sampled nonblank telemetry-stream identifiers with replacement and retained all associated gain-valid days; a repeatedly sampled identifier contributed its complete block repeatedly. No 5000-row cap was applied. This preserves within-stream dependence while retaining a day-weighted descriptive estimand, but it does not model additional apiary-, year- or management-level dependence. Additional diagnostics used ambient-temperature bins, within-stream median contrasts, year-specific effects, minimum total stream-day thresholds of 10, 20, 30, 45, 60 and 90 days, exclusion of the top 0%, 1%, 2%, 5% and 10% of streams by contribution, event thresholds from 0.10 to 1.00 kg, and random stream subsampling at 50%, 65% and 80% for 100 repetitions per fraction (seed 42). Selection effects were assessed by comparing the 110 retained streams with both the 98 role-eligible low-confidence streams and all nonretained streams with usable mass data.

### 2.10. Grouped Predictive Diagnostics

The forecast origin was 00:00 in Europe/Warsaw on the target calendar date. Features used exact calendar-day lags of 1, 2, 3 and 7 days; a missing prior date produced a missing feature. Availability was checked against the origin in UTC, preserving daylight-saving offsets. An hourly bin was available only after its end. The centred three-point mass smoother required the next observed bin, and the neighbouring-jump suppression used for positive-rate hours required a further bin. A derived value was withheld if any required timestamp was later than the forecast origin. In particular, 378 otherwise non-missing lag-1 daytime changes and 491 lag-1 positive-rate hour counts were withheld. Rebuilding features after truncating the hourly archive at three forecast origins passed 228 feature-level prefix-invariance checks (Supplementary Data S1).

For prediction, overnight mass change indexed by source day *k* was defined as morning mass on *k* minus evening mass on k−1; completed 24 h change was morning mass on *k* minus morning mass on k−1. Thus, their lag-1 versions end on the morning before the target date. This differs from the retrospective night-change descriptor in Section 2.7, whose interval extends to the next morning. The original target values and 20,034 modelling rows were retained; source history was reconstructed from the hourly records even when a source day’s prediction target was missing.

The biological role assignments in Section 2.8 remain retrospective. For predictive features only, role scores were recomputed from strictly earlier active-season days of each stream, using the same score formulas and requiring at least 30 earlier days with finite candidate-channel and ambient daily means and ranges. The within-day eight-concurrent-hour criterion of the retrospective audit was not imposed on this expanding predictive mapping. The source day’s temperatures and thermal state used this past-only mapping. Internal descriptors were missing and the state was unknown until a qualifying internal/core-like mapping was available. This construction removes future information from the feature mapping, but the modelling population remains the retrospectively selected cohort; it is not a prospective cohort-selection validation.

All models also contained sine and cosine of target-day day of year. Predictive comparisons used the following feature families:

1. **Weather history**: A total of 29 fixed daily weather descriptors at each lag (116 history variables plus two calendar terms). These include temperature, precipitation, radiation, sunshine, wind, humidity, dew point, vapour-pressure deficit, evapotranspiration, temperature range, growing-degree-day indices and fixed-threshold weather indicators;
2. **BeeHUB sensor history**: Fourteen daily microclimate and acoustic descriptors at each lag, plus the categorical past-only thermal state (58 numeric and four categorical variables including calendar terms);
3. **Operational weight history**: Daytime mass change, hours with positive mass rate, completed overnight change and completed 24 h change at each lag (18 numeric variables including calendar terms);
4. **All operational history**: All preceding variables (190 numeric and four categorical variables).

The two calendar terms are shared. Thus, combined non-weight sensor–weather history contains 174 numeric and four categorical variables. The corrected manifest contains no duplicate row-shift weather columns and no sample-wide seasonal radiation-quantile flag. Weather observations were joined by location and exact calendar date. Their observation days precede the forecast origin, but historical weather-product release times were not available: this is an observation-time retrospective benchmark, not a verified real-time weather-feed evaluation. Exact feature lists and availability checks are provided in Supplementary Data S1.

A separate same-day contextual analysis was also performed. Within each season, it used the same canonical 75/25 stream-grouped split as the strict temporal analysis. Weather-only variants contained either 30 current-day weather variables plus two calendar terms (32 numeric variables) or current, lagged and rolling weather variables plus the calendar terms (104 numeric variables). The non-weight BeeHUB family contained 14 current-day microclimate and acoustic variables, two calendar terms and one categorical inferred thermal-state variable (16 numeric plus one categorical variable). Their combined representation contained 118 numeric and one categorical variable. Current-day and future mass-change variables were excluded. This analysis therefore describes contemporaneous contextual discrimination and is not presented as a day-ahead forecast. Supplementary Table S8 summarises these descriptive results; the complete feature lists and estimator-specific metrics are provided in Supplementary Data S1. This contextual analysis retains retrospective role mappings and weather preprocessing and is separate from the corrected forecast pipeline.

Numeric missing values were median-imputed and categorical missing values were mode-imputed using only the training partition relevant to each fitting step: the inner-training subset during cutoff selection and the complete outer-training partition during final refitting. Numeric variables were standardised to zero mean and unit variance, and categorical variables were one-hot encoded with unseen categories ignored. Logistic regression used max_iter=3000, class_weight=balanced and seed 42. Histogram gradient boosting used 250 iterations, a learning rate of 0.05, 31 maximum leaf nodes and seed 42, with early stopping disabled so all 250 iterations were fitted. The primary HGB analysis was unweighted. HGB with balanced sample weights was additionally evaluated as a sensitivity model; focal loss was not tested. Hyperparameters were fixed, and no grid or Bayesian hyperparameter search was performed.

For each outer split, threshold-independent metrics were calculated from the untouched test-set probabilities. An operational probability cutoff was selected on a separate stream-grouped validation subset constructed only from the outer training data by maximising balanced accuracy. The model was then refitted on the complete outer training partition and evaluated on the outer test set. Metrics at both the fixed cutoff of 0.5 and the training-selected cutoff were retained. Imputation, scaling and encoding were fitted anew on the inner-training subset during cutoff selection and on the complete outer-training partition during refitting. No test-set outcomes were used to select the operational cutoff.

The corrected predictive analyses were executed using Python 3.12.14 and scikit-learn 1.8.0 [52]. Histogram gradient boosting follows the general additive boosting framework introduced by Friedman [53]. The exact executed feature manifest and software environment are provided in Supplementary Data S1.

Within each season, GroupShuffleSplit was repeated 50 times, with 25% of nonblank telemetry-stream identifiers assigned to the test set in each split. No stream identifier occurred in both training and test data in any split. The seed-42 partition was retained as a canonical split for exact test counts, calibration curves, paired comparisons and whole-stream uncertainty intervals. Means and standard deviations across the 50 outer splits quantify sensitivity to the selected train–test partition. Grouping prevents row-level leakage from a recorded stream; because device–colony assignment histories were unavailable, it does not establish generalisation to previously unseen biological colonies [54].

ROC AUC, average precision and Brier score were calculated for every split. Average precision was included because precision–recall evaluation is particularly informative when class frequencies are unequal [55]. Sensitivity, specificity, precision, F1 and balanced accuracy were calculated at both the fixed 0.5 cutoff and the training-selected cutoff. For the canonical split, 95% confidence intervals for ROC AUC, average precision and Brier score were obtained from 2000 whole-stream cluster-bootstrap resamples of the held-out identifiers. These intervals are conditional on the canonical split; the standard deviation across 50 splits describes a separate source of uncertainty. Reliability diagrams used eight quantile-based bins on the canonical held-out split. No post hoc probability-calibration model was fitted (Supplementary Figure S1).

A persistence baseline used previous-day daytime mass change as a continuous score on the same held-out rows; 0.3 kg was its operational classification threshold. Missing lag-1 values were filled with the corresponding outer-training median to preserve identical rows for model comparison, and a complete-case sensitivity analysis was also performed. The added value of the full unweighted-HGB model was quantified by paired whole-stream bootstrap differences in ROC AUC and average precision on the canonical split.

Because verified apiary identifiers were unavailable, an additional sensitivity analysis repeated 50 grouped splits using rounded coordinate pairs as location-grouping proxies and the primary unweighted-HGB specification, with no rounded-location overlap between training and test data. Rounded coordinates are not verified apiary identifiers; this analysis therefore reduces but cannot eliminate concern about shared-location or shared-management dependence (Supplementary Table S2).

Table 6 reports the exact canonical partitions used for the split-conditional diagnostics.

Feature importance was computed for the canonical unweighted-HGB test split by permutation with three repeats, seed 42 and ROC AUC as the scoring function. All 899, 1318, 1530 and 1549 test stream-days were used in early spring, May–June, summer and autumn, respectively. Because feature groups and two algorithms were compared on the same held-out split, their differences are descriptive model comparisons rather than an independently tuned-and-tested model selection exercise. Accordingly, permutation importance was interpreted as a model- and split-dependent diagnostic, rather than as evidence of causal feature relevance [56].

### 2.11. Reporting Strategy

Exact values are reported from the frozen revision outputs. Whole-stream bootstrap intervals account for repeated days within a telemetry stream but not for all higher-level clustering. Repeated-split estimates and canonical- split bootstrap intervals address different sources of uncertainty and are reported separately. Correlations, same-day model-family comparisons and the remaining robustness analyses in the Supplementary Material are descriptive; they were not interpreted as causal effects or as a family of confirmatory hypothesis tests.

## 3. Results

### 3.1. Weather Matching Provided Broad Coverage and an External Temperature Consistency Check

**Figure 5:**
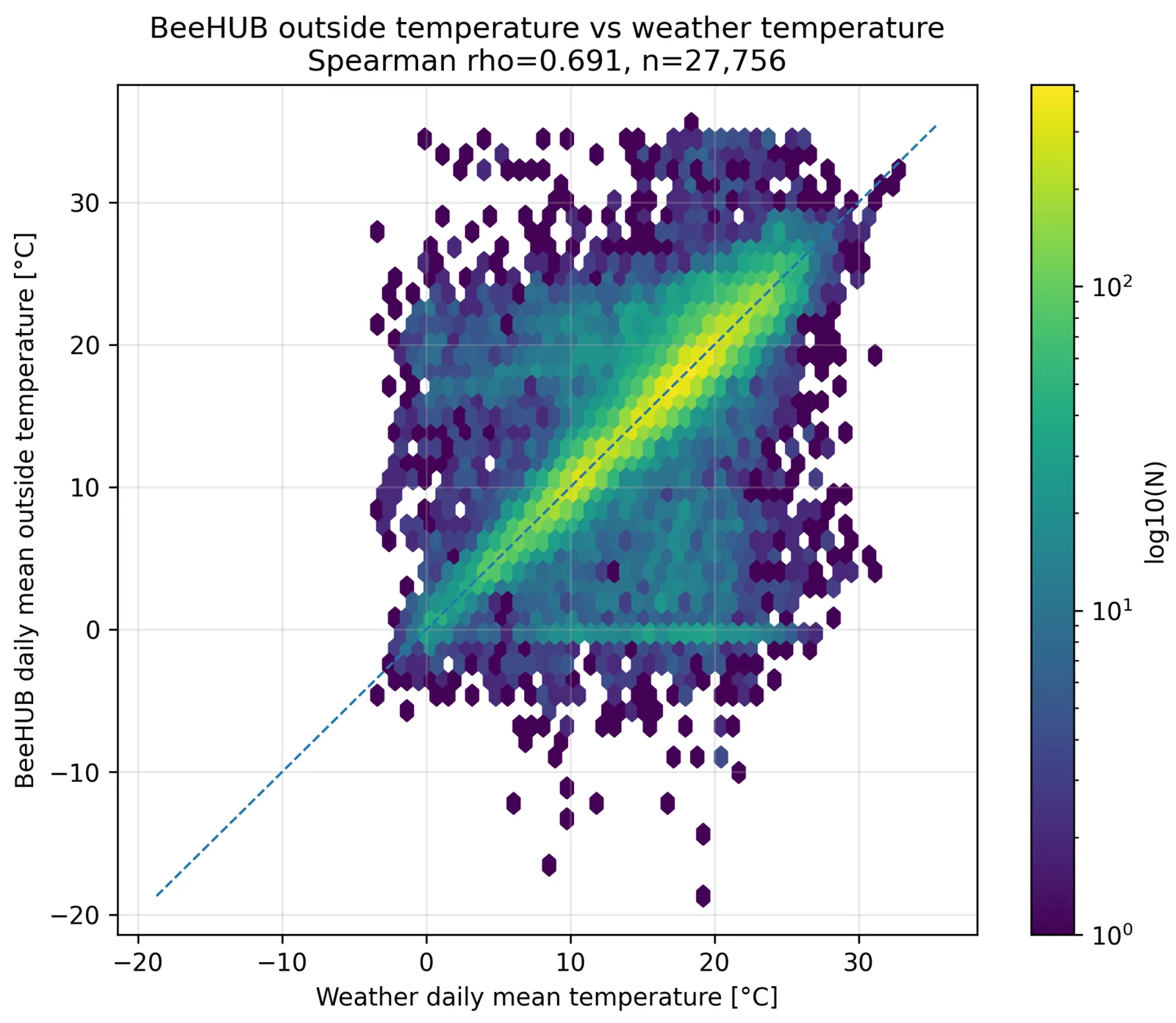
Comparison of daily mean ambient temperature measured by BeeHUB with location-matched meteorological mean air temperature. The comparison is used as a cross-source consistency diagnostic and should not be interpreted as a formal field calibration of the BeeHUB temperature sensor.

Location-matched weather was available for 27,756 of 35,004 role-corrected active-season stream-days (79.3%). Seasonal coverage was similar across the four analysed periods, ranging from 78.8% to 80.2%. As an archive-level consistency check, daily BeeHUB ambient-temperature measurements were compared with location-matched meteorological mean air temperature (Figure 5). The comparison supports the use of matched weather as contextual environmental information. Differences between the two measurements are expected because BeeHUB probes represent local microsite conditions, whereas the meteorological data represent spatially modelled weather conditions at rounded coordinates.

### 3.2. Temperature Channels Showed Heterogeneous Behavioural Roles

**Figure 6:**
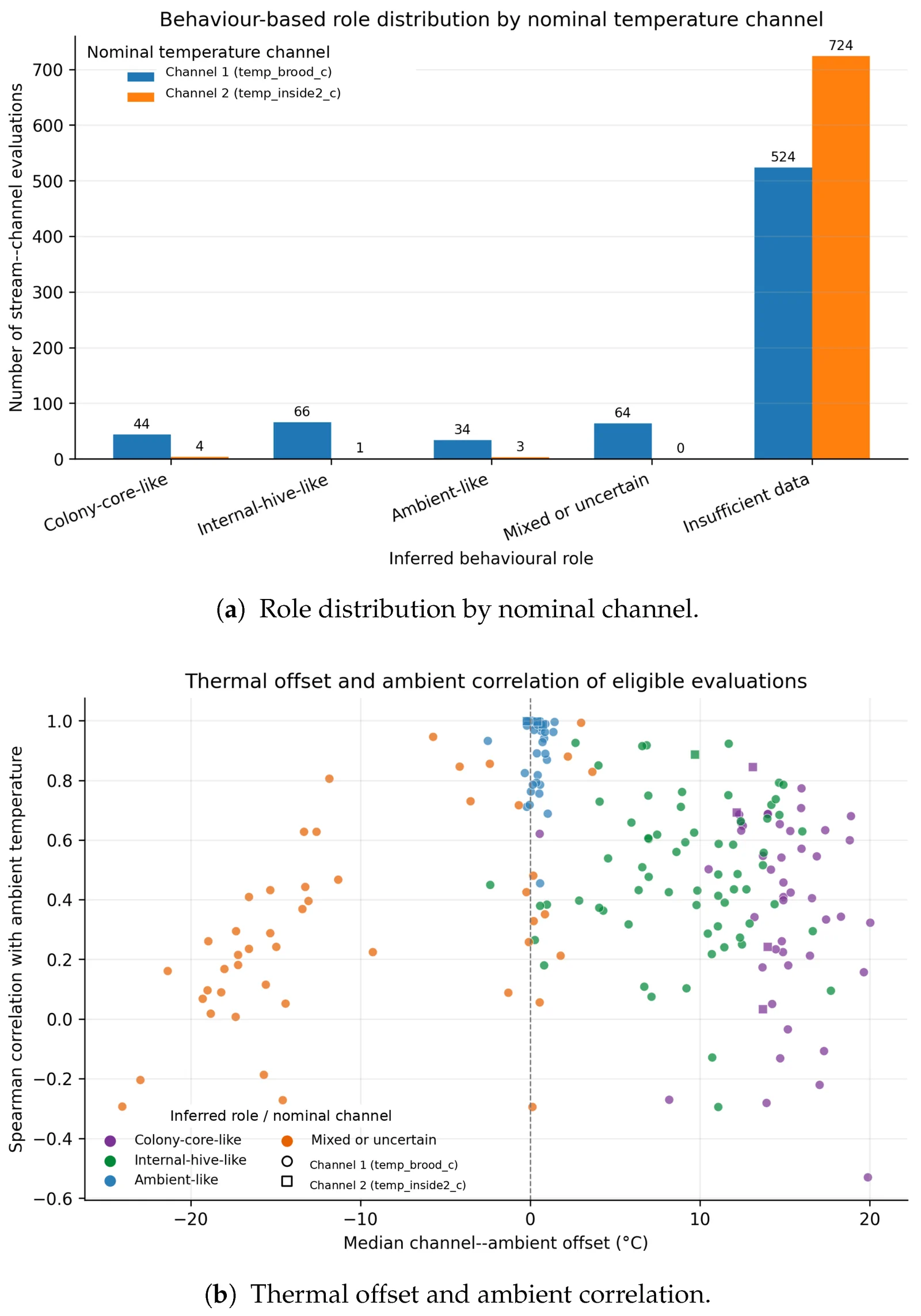
Behaviour-based interpretation of the two nominal temperature channels after exclusion of the blank identifier. (**a**) Inferred role distribution by nominal temperature channel; (**b**) thermal-offset–ambient-correlation diagnostic space for eligible stream–channel evaluations. Reader-facing channel labels are used in the legends, with the original database field names retained in parentheses for traceability. Nominal channel identity did not uniquely determine inferred role. The score margin and confidence tiers are heuristic, and the assignments were not validated against technician-recorded placement.

The raw columns temp_brood_c and temp_inside2_c did not map one-to-one to inferred roles (Figure 6). After exclusion of the blank identifier, temp_brood_c comprised 524 insufficient-data, 64 mixed-or-uncertain, 66 internal-hive-like, 44 colony-core-like and 34 ambient-like stream–channel evaluations. The corresponding counts for temp_inside2_c were 724, 0, 1, 4 and 3. Thus, both nominal columns exhibited more than one behavioural role among evaluable streams, although temp_inside2_c had sufficient joint channel–ambient coverage in only eight streams. Across identifiers, 732 nonblank streams entered the role audit, 208 had at least one candidate channel with at least 30 valid joint channel–ambient days, and 110 received a high- or medium-confidence internal-channel mapping. These 110 streams contributed to the role-corrected biological analysis. The former total of 733 evaluations per nominal channel resulted from a blank-identifier group; its two role rows and 328 daily biological rows were removed before the revised downstream analyses.

The score margin was used only as a heuristic ambiguity indicator, not as a probability or statistical confidence interval. Among the 216 eligible channel evaluations, 38 (17.6%) had a top-versus-second score margin below 0.10. Among the 110 selected streams, 27 (24.5%) had a selected-sensor margin below 0.10. Sixty-seven selected stream–sensor pairs were evaluable in at least two separate years, and 41 (61.2%) received different annual role labels. Such differences cannot distinguish sensor repositioning from changes in colony behaviour, ambient conditions or data availability, but they show that the time-invariant assignment is a material simplification. Across the prespecified role-parameter perturbations, eligible-channel role agreement with the primary rule ranged from 93.1% to 100%, selected-sensor agreement was at least 99.5%, and 103–114 streams were retained (Supplementary Tables S3 and S4).

Selection into the role-corrected biological subset was not neutral. The comparison used March–October days within the analytical interval and the same accepted daytime-change range of −5 to 10 kg in all groups. The 110 retained streams contributed 28,544 gain-valid days, with a median daytime mass change of 0.100 kg and an event rate of 33.9%, compared with 20,790 days, 0.050 kg and 28.4% in the 98 role-eligible low-confidence streams. Across 2000 whole-stream bootstrap resamples, the retained-minus-eligible median difference was 0.050 kg (95% CI −0.050 to 0.100), and the event-rate difference was 5.48 percentage points (95% CI 0.80 to 10.28). Of all 622 nonretained role-audit streams, 514 contributed usable mass data within this window (130,659 days). Relative to those streams, the median difference was 0.000 kg (95% CI −0.050 to 0.035) and the event-rate difference was 0.36 percentage points (95% CI −3.68 to 4.42). Maintenance quality was not recorded and cannot be inferred. Biological results are conditional on the selected 110-stream subset (Supplementary Table S5).

**Figure 7:**
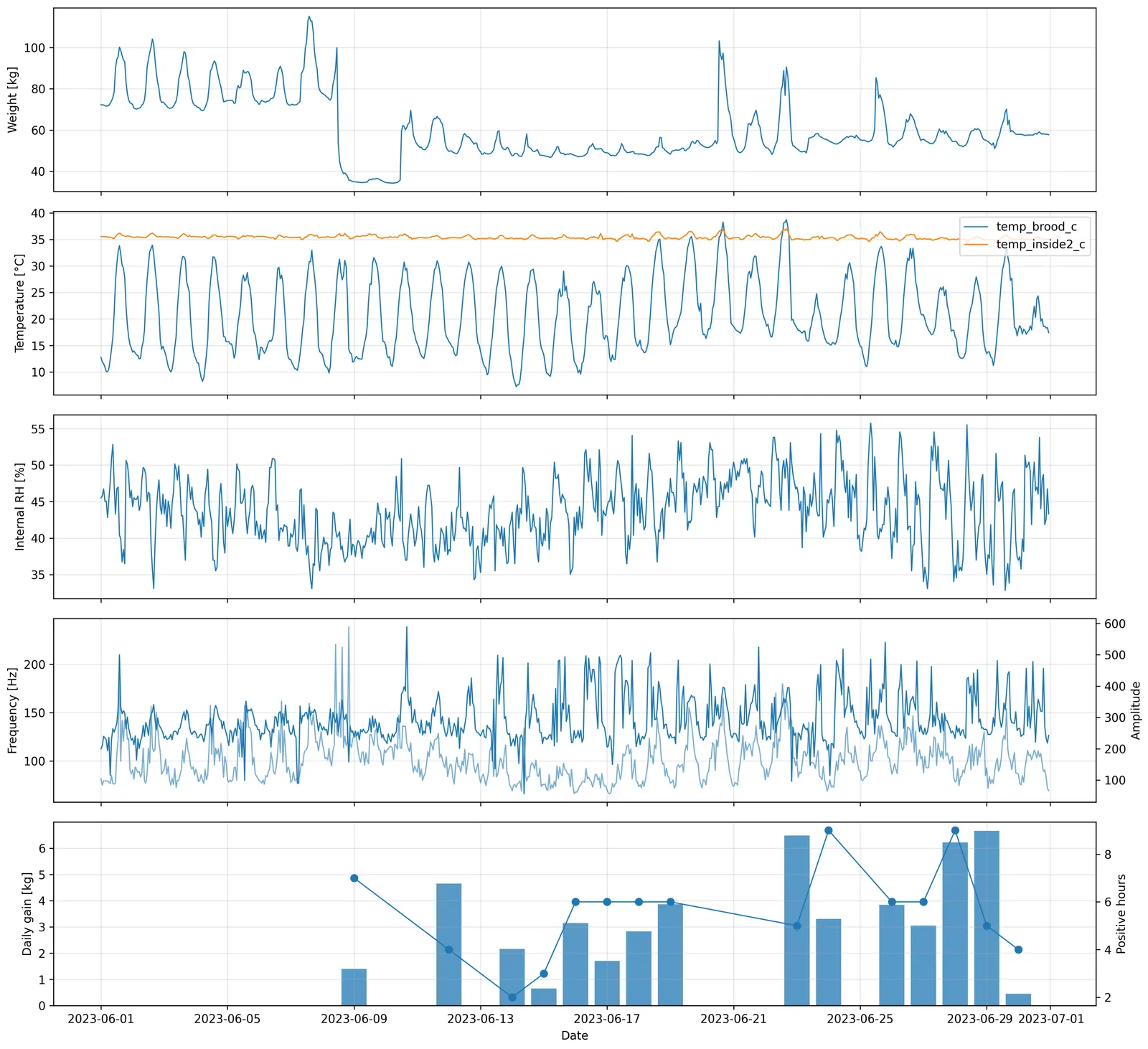
Representative BeeHUB telemetry trace illustrating temperature-channel heterogeneity. From top to bottom, the panels show hive mass; the two nominal temperature channels; internal relative humidity; acoustic frequency and uncalibrated amplitude in relative device units; and daytime mass change with hours of positive mass rate. The original temperature labels temp_brood_c and temp_inside2_c identify the more diurnally variable and relatively stable signals, respectively. In the lower panel, “Daily gain” denotes daytime mass change and “Positive hours” denotes hours with positive mass rate. No event-specific markers or verified management labels are assigned. This example is illustrative, was not used for target annotation, and does not validate physical sensor placement or the causes of mass changes.

Figure 7 provides a representative deployment-level example of the semantic heterogeneity observed in the archive. In this example, one nominal temperature channel showed strong diurnal variation, whereas the second remained close to brood-compatible temperatures. The trace is illustrative and was not used as a technician-verified sensor-placement label.

**Figure 8:**
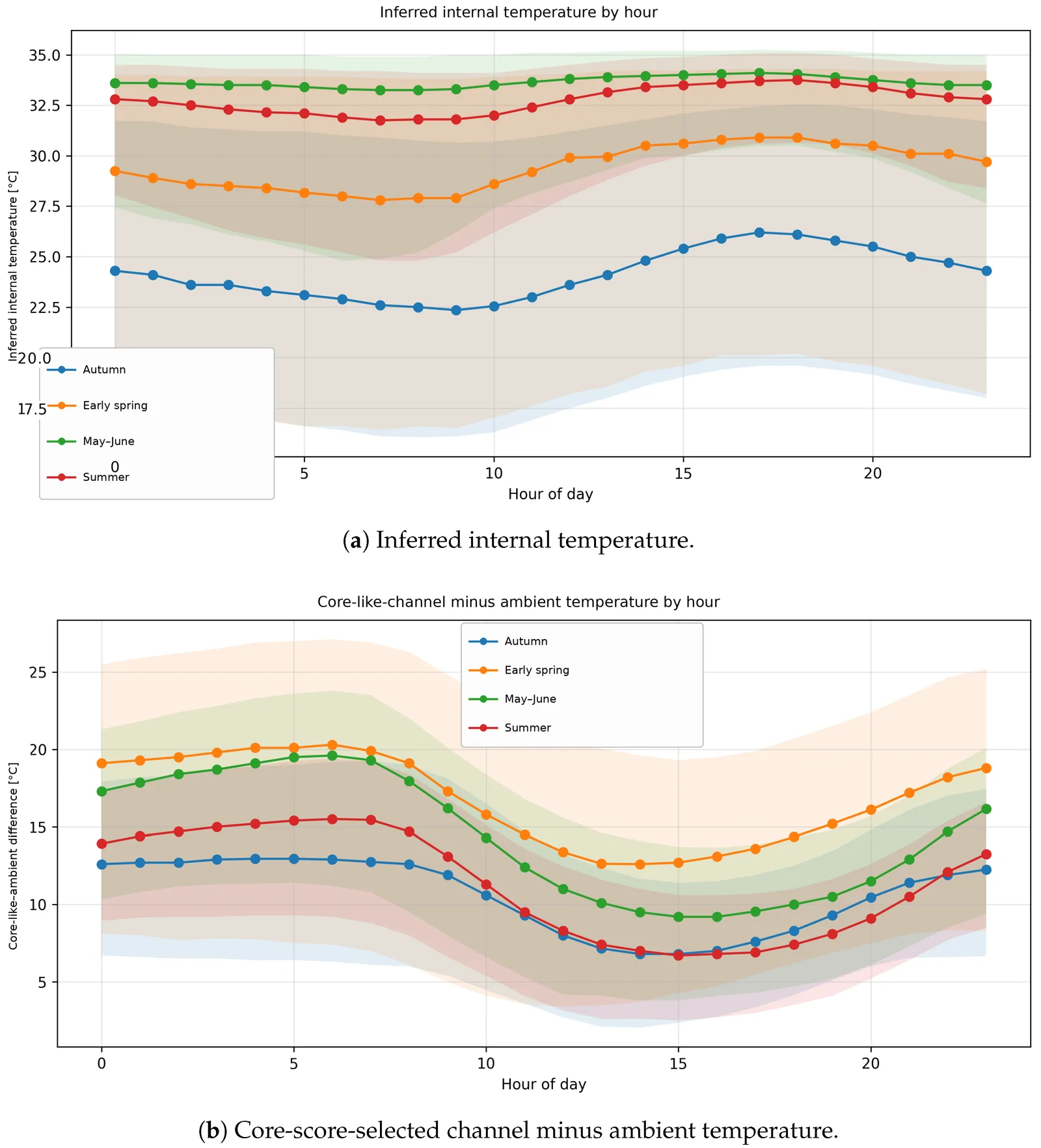
Median diurnal temperature profiles by calendar period after behaviour-based channel interpretation. (**a**) Selected inferred internal channel; (**b**) independently core-score-selected channel minus ambient temperature. Both panels use streams with high- or medium-confidence internal-channel mappings. Lines show medians and shaded bands the interquartile range of pooled available hourly observations. Selection by core score does not require the assigned role colony-core-like. The two selection rules may identify the same or different channels.

The inferred internal-channel profile remained warm across the principal active calendar periods, while the core-score-selected channel profile showed a positive difference from ambient temperature across the daily cycle (Figure 8). These patterns provide biological plausibility for the behaviour-based interpretation, but they are conditional on selected streams and do not independently verify exact sensor location or colony condition.

### 3.3. Warm-Stable Days Were Most Frequent in May–June and Summer

**Figure 9:**
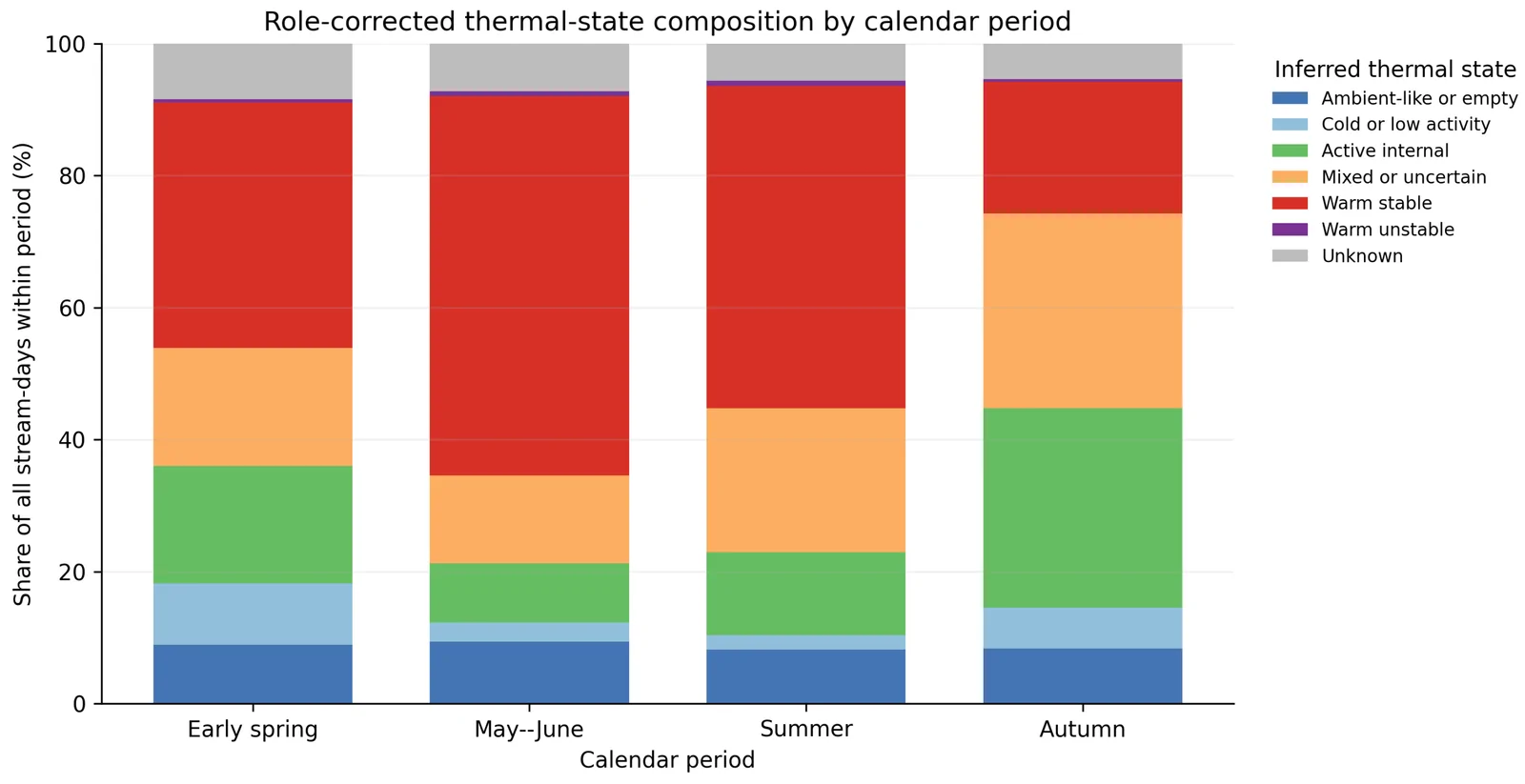
Composition of inferred thermal states by calendar period after removal of the blank identifier. Plotted percentages use all corrected stream-days in each period, including the unknown state. The text additionally reports warm-stable proportions after excluding unknown-data days.

Thermal-state composition changed markedly by calendar period (Figure 9). Excluding unknown-data days, the warm-stable state accounted for 61.9% of classified May–June stream-days (5070 of 8187) and 51.8% in summer (4906 of 9472), compared with 40.6% in early spring (2372 of 5842) and 21.1% in autumn (1947 of 9230). With unknown-data days retained in the denominator, the corresponding proportions were 57.5%, 48.9%, 37.2% and 20.0%.

During the calendar-defined May–June period, warm-stable days had a median inferred internal temperature of 34.8 °C, a median thermal buffer of 17.0 °C, a median daytime mass change of 0.35 kg and an event rate of 52.5% among 4372 gain-valid days from 96 streams. These are conditional, descriptive associations within the selected stream subset; they neither identify a biological mechanism nor establish verified foraging intake. Unknown and warm-unstable days had numerically similar event rates but were represented by only 158 and 48 gain-valid days, respectively, and therefore do not support a claim that warm-stable days had the highest rate across all states.

Table 7 reports the outcome denominator and descriptive mass-change result for every inferred May–June state.

### 3.4. Warm-Stable Associations Persisted Under Whole-Stream Resampling

In the calendar-defined May–June period, whole-stream bootstrap comparisons showed higher warm-stable median mass change and event rate than the four common comparison states shown in Table 8. The contrast with the small warm-unstable group was imprecise and included zero. These are day-weighted, descriptive associations conditional on inferred state and the selected streams, not causal estimates.

The May–June event-rate contrasts were robust across operational mass-change thresholds from 0.10 to 1.00 kg. Warm-stable minus comparison-state differences were positive at all ten thresholds and their whole-stream bootstrap intervals excluded zero for active-internal (range 10.0–18.3 percentage points), mixed/uncertain (7.9–11.1), ambient-like/empty (16.2–32.4) and cold/low-activity (21.5–33.8) states. By contrast, warm-stable minus warm-unstable differences were negative at every threshold (−18.4 to −1.7 percentage points), with substantial uncertainty because the warm-unstable sample was small. Restriction to high-confidence mappings or selected-score margins of at least 0.10 preserved the positive direction for the three principal comparisons. Restriction to the 26 annually stable multiyear mappings, or the 20 mappings that were both high-confidence and stable, preserved most point-estimate directions but widened intervals and caused some active/mixed median-change intervals to include zero (Supplementary Tables S4 and S7).

**Figure 10:**
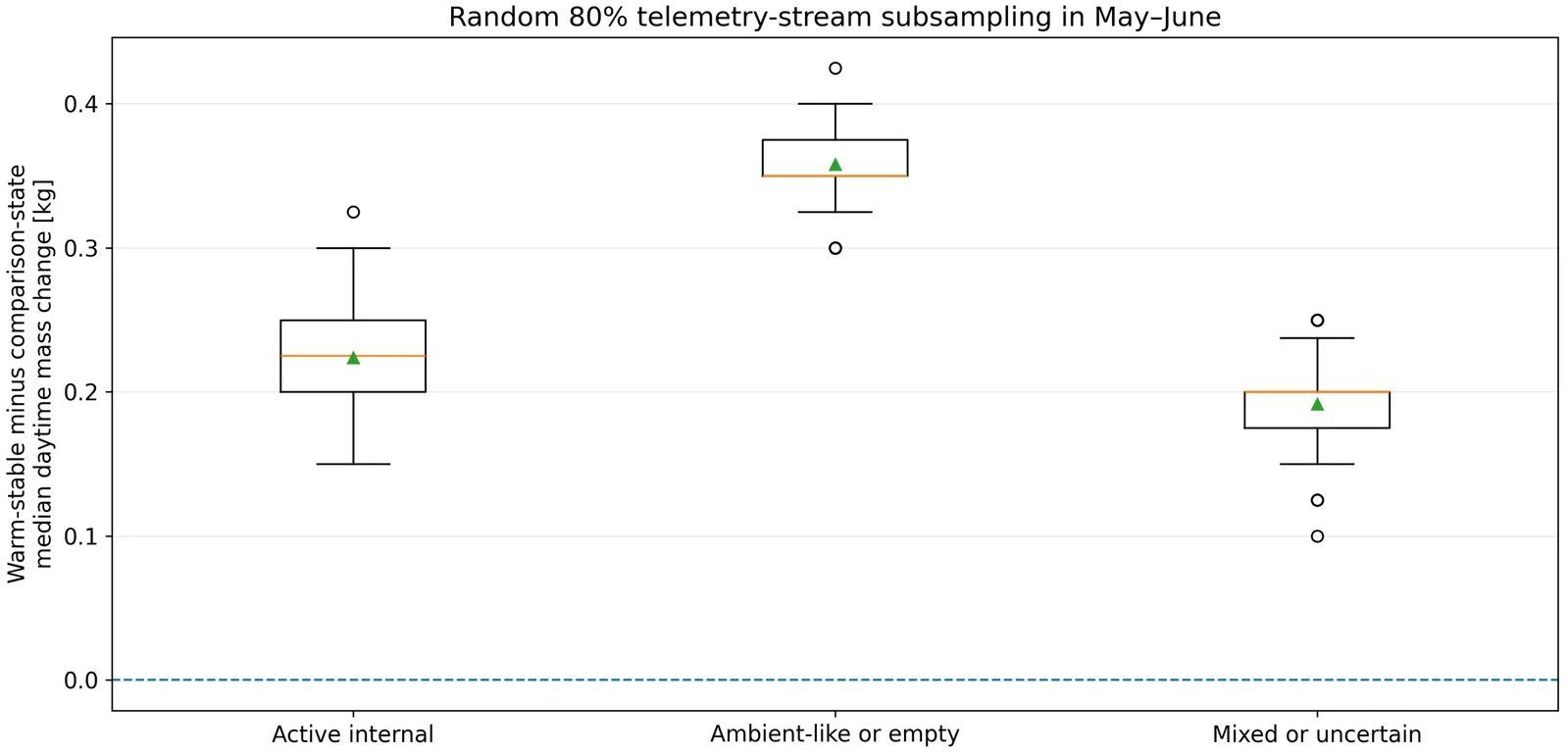
Random stream-subsampling sensitivity for the calendar-defined May–June period, using 80% of streams without replacement in each of 100 repetitions. The vertical scale is the warm-stable minus comparison-state median daytime mass change. This is a stability diagnostic, not an independence-adjusted confidence interval.

Across the 50%, 65% and 80% random stream-subsampling fractions, each of the three principal comparisons contributed 300 runs (100 per fraction), and the median-change contrast was positive in every run. Figure 10 shows the 80% fraction; the remaining prespecified diagnostics are reported in Supplementary Table S7.

**Figure 11:**
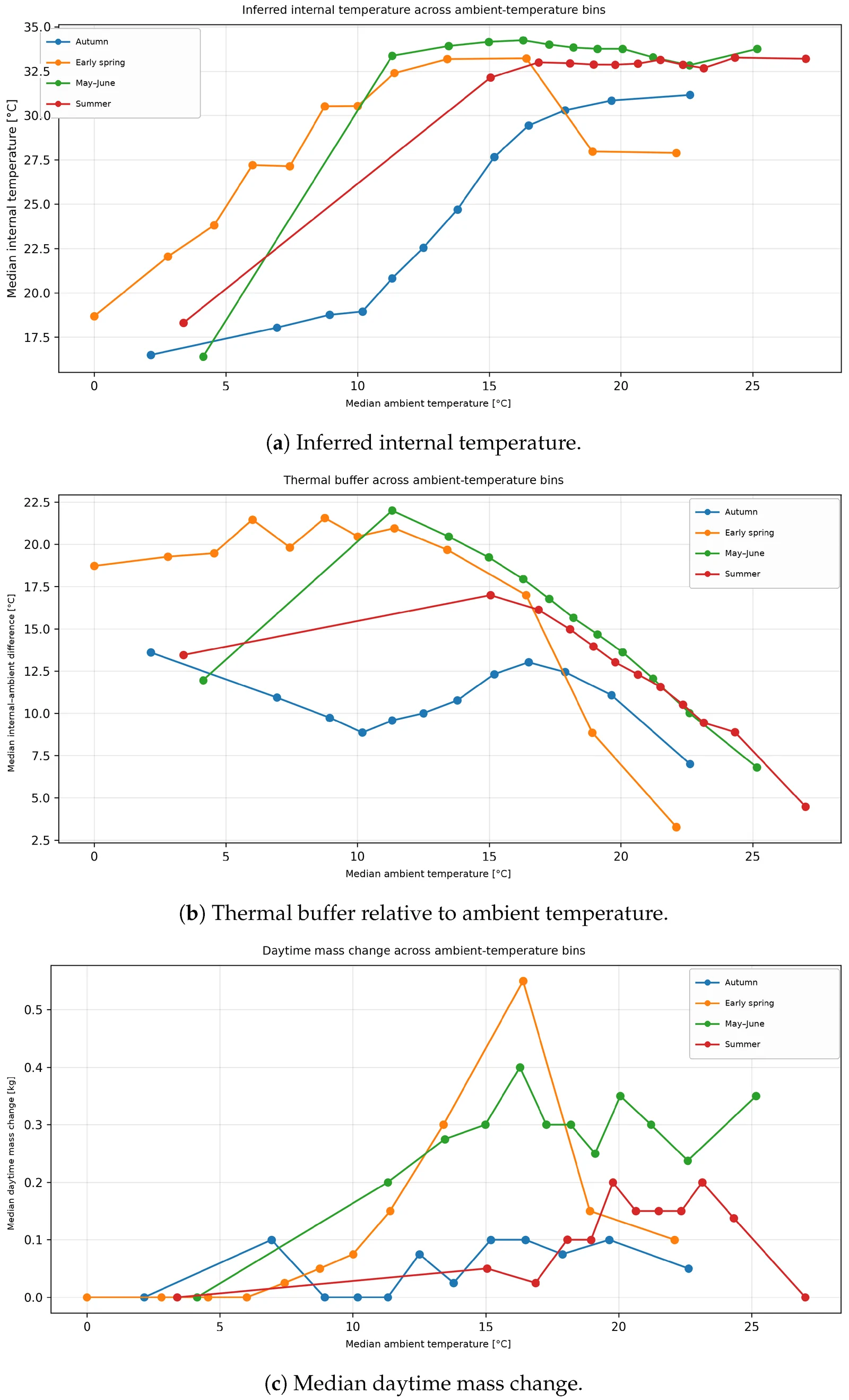
Descriptive response curves across median ambient-temperature bins and seasons. (**a**) Inferred internal temperature; (**b**) internal-to-ambient thermal buffer; and (**c**) median daytime mass change. The temperature panels provide context for interpreting the warm-stable thermal phenotype, whereas daytime mass change represents the operational weight-derived outcome. Observations are repeated stream-days, and the curves were not adjusted for stream, rounded-location group, year, colony strength, floral resources or management. They should therefore not be interpreted as causal effects of ambient temperature.

The descriptive bin-level patterns underlying the ambient-temperature diagnostic are shown in Figure 11. The inferred internal signal remained relatively warm and buffered across a broad range of ambient conditions, whereas daytime mass change showed a more season-dependent response.

### 3.5. Non-Weight Context and Operational History Answered Different Questions

**Figure 12:**
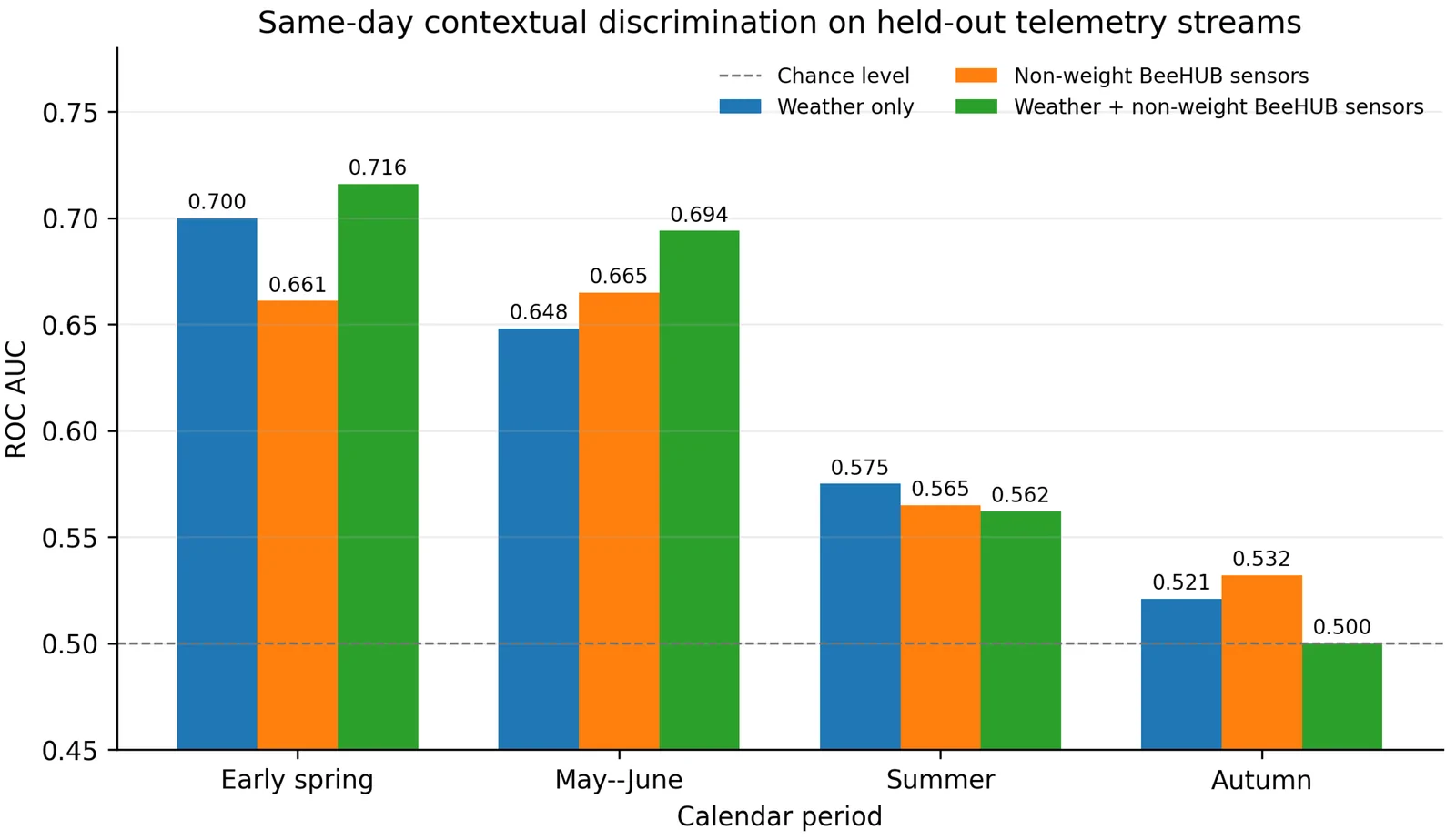
Same-day contextual discrimination on the canonical held-out- stream split. Current-day and future mass-change variables were excluded. For each feature family and season, the displayed value is the better result from the two prespecified estimators and is therefore descriptive rather than a nested model-selection estimate. Exact estimator-specific ROC AUC and average-precision values for both estimators are provided in Supplementary Data S1; Supplementary Table S8 summarises the displayed comparisons.

In the separate same-day contextual analysis, the best weather-only, non-weight BeeHUB-only and combined models achieved ROC AUC/AP of 0.700/0.463, 0.661/0.444 and 0.716/0.480 in early spring; 0.648/0.576, 0.665/0.601 and 0.694/0.627 in May–June; 0.575/0.372, 0.565/0.351 and 0.562/0.369 in summer; and 0.521/0.255, 0.532/0.254 and 0.500/0.235 in autumn, respectively (Figure 12). Current-day and future mass-change variables were excluded, and the canonical held-out-stream split was used. For compactness, the values above are the better result from the two prespecified estimators within each season and feature family. Because that choice was based on test-set performance, these comparisons are descriptive rather than nested model-selection estimates. They describe contemporaneous context, not strict day-ahead forecasting; Supplementary Table S8 summarises the selected results, and complete estimator-by-family results are provided in Supplementary Data S1.

Strictly lagged operational history provided stronger discrimination. Across 50 grouped splits, the corrected primary unweighted-HGB all-history model achieved mean ROC AUCs of 0.748, 0.809, 0.797, 0.783 and mean average precisions of 0.624, 0.793, 0.712, 0.611 in early spring, May–June, summer and autumn, respectively (Table 9). Corresponding between-split ROC AUC standard deviations were 0.020, 0.018, 0.026, 0.031. Balanced HGB had similar discrimination and higher mean Brier scores in three of four periods; unweighted HGB remained the primary specification. Training-selected cutoffs improved the sensitivity–specificity balance relative to 0.5, particularly in early spring and autumn. They did not improve held-out balanced accuracy in every model or period (Supplementary Table S12), and are not universal deployment thresholds.

Repeated predictive results and model comparisons are summarised in Supplementary Table S9; split-level results and bootstrap outputs are provided in Supplementary Data S1.

The repeated full-model and persistence results are compared directly in Table 10.

The persistence baseline captured a substantial fraction of discrimination, confirming the autoregressive character of the task. Nevertheless, the full model had higher canonical point estimates for both metrics. Paired whole-stream intervals excluded zero for the AUC difference in early spring, May–June and summer, but not in autumn (−0.003 to 0.095). AP-difference intervals were positive in all four periods. On rows with an observed lag-1 gain, the full model also retained higher mean ROC AUC and average precision across splits (Supplementary Data S1).

Canonical-split Brier scores for the primary unweighted-HGB model were 0.167, 0.186, 0.147, 0.144 in early spring, May–June, summer and autumn, respectively; reliability diagrams are shown in Supplementary Figure S1. These internal diagnostics do not establish calibration at a new deployment. In the 50-split rounded-location sensitivity analysis, mean ROC AUC/AP was 0.749/0.616, 0.804/0.787, 0.798/0.710, 0.784/0.606 across the four periods. These results provide a further check on spatial grouping, but rounded coordinates remain imperfect apiary proxies (Supplementary Table S2).

Matched feature-family reruns confirmed the importance of mass history. Weight-only HGB achieved mean ROC AUCs of 0.745, 0.783, 0.792, 0.785. In particular, its autumn AUC was similar to the full model’s; the added value of non-weight measurements was not uniform across periods. Weather-only and non-weight sensor-history results, using the same partitions and corrected availability rules, are reported in Supplementary Table S11.

Predictive event-threshold sensitivity used 20 common grouped partitions at each cutoff from 0.10 to 1.00 kg (Supplementary Figure S2). The full model retained higher mean ROC AUC than persistence in all 40 season–threshold comparisons. These are descriptive comparisons; changing the event cutoff also changes event prevalence and the prediction task, and the test results were not used to choose the operational 0.3-kg definition.

**Figure 13:**
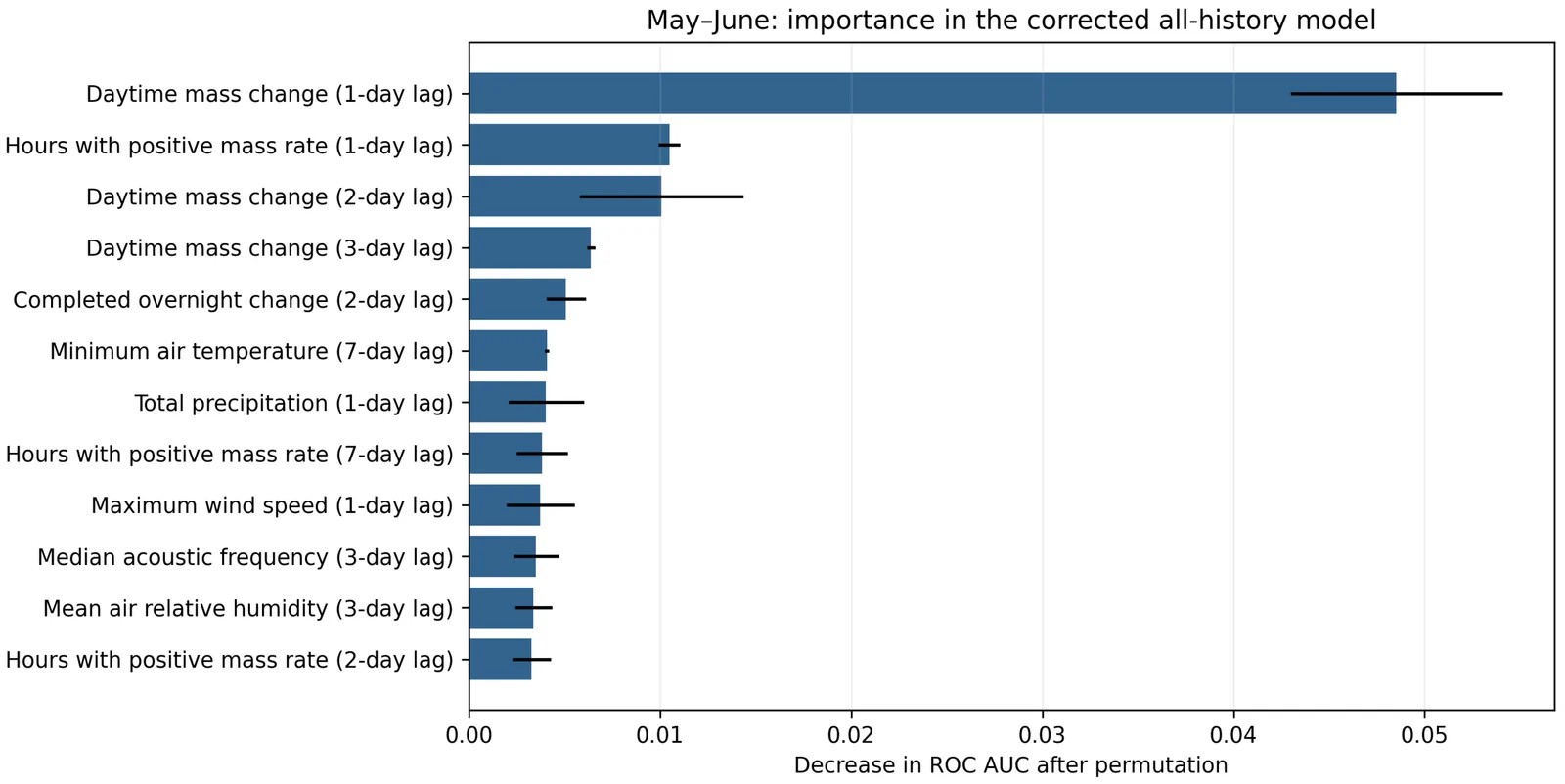
Permutation importance for the canonical May–June unweighted-HGB all-history classifier. Bars show the mean decrease in ROC AUC across three permutation repeats, and whiskers indicate one standard deviation across those repeats. They do not represent confidence intervals obtained by resampling telemetry streams. All 1318 held-out stream-days were used, with no row subsampling. Feature suffixes denote exact calendar-day lags. Importance is model- and split-dependent and is not causal evidence.

Permutation importance, recomputed for the corrected canonical May–June model, ranked previous-day daytime mass change first (mean ROC AUC decrease 0.049), followed by previous-day hours with positive mass rate (0.010) and two-day-lagged daytime mass change (0.010; Figure 13). This supports an autoregressive interpretation of the fitted model. Permutation importance is conditional on that model, its correlated features and the selected test streams; it is not evidence of nectar availability or a biological mechanism.

## 4. Discussion

### 4.1. Sensor Semantics Are Part of the Scientific Problem

The first contribution is methodological: temperature-channel identity cannot be assumed from a database column name in a heterogeneous field deployment. The observed overlap of inferred roles across raw columns is consistent with the pattern expected when placement and installation vary, although colony condition, ambient exposure and data coverage may produce similar behavioural differences. Previous experimental work has shown that proximity to the hive centre and brood area changes the relationship between temperature and colony strength [6]. Treating semantic uncertainty explicitly is therefore preferable to pooling raw columns under a fixed biological label.

The uncertainty analyses qualify this conclusion. Twenty-seven of the 110 selected streams (24.5%) had a top-versus-second role-score margin below 0.10, and 41 of 67 selected mappings evaluable in multiple years changed their annual behaviour-based role. Annual disagreement is compatible with sensor repositioning, but can also reflect genuine changes in colony thermoregulation, ambient exposure or data coverage. Because the selected channel supplies both inferred internal temperature and the thermal buffer, a mapping error can propagate directly into daily state assignment and then into state-specific mass-change contrasts. Restricted analyses using stable or less ambiguous mappings largely preserved the directions of the principal contrasts but were less precise. The biological results should therefore be read as conditional on the mapping rule, not as validated placement-specific effects.

The evidence nevertheless supports *role inference*, not role validation. Behavioural consistency with thermal buffering makes an internal interpretation plausible, but manual placement logs or controlled reference sensors are needed to quantify errors. A future validation study should compare time-segmented inferred roles with technician-verified positions and sensor-replacement logs, report a confusion matrix, and determine whether change-point detection can identify within-deployment sensor relocation.

### 4.2. Thermal State Provides Biological Context, Not a Causal Mechanism

Within the selected 110-stream subset, warm-stable days in the May–June calendar period combined a brood-compatible inferred internal temperature, strong buffering and greater median mass increase than active-internal, mixed/uncertain, ambient-like/empty and cold/low-activity days. The sparse warm-unstable state did not show the same contrast, and unknown days had substantial outcome missingness. Field experiments using the same broader smart-hive monitoring concept have likewise shown that hive thermal behaviour can be quantified from continuous IoT telemetry when evaluating changes in the physical hive environment [18]. These patterns remain associative: colony strength, brood area, floral resources, weather, management and sensor placement can jointly affect temperature and mass, and no colony-level field observations were available to identify a mechanism. The retained streams also had a moderately higher event rate than the 98 role-eligible low-confidence streams (33.9% versus 28.4%), demonstrating selection at the mapping stage, although comparison with all nonretained weight-bearing streams did not indicate simple enrichment for the most active deployments. The thermal phenotype is therefore a conditional contextual or triage signal, not evidence that thermal state causes resource collection or that the event is verified nectar intake.

The strong seasonal variation is equally important. A state that is common and informative in the May–June calendar period may not have the same prevalence or meaning in autumn. Thresholds and expected baselines should be period-aware, and performance should be reported separately rather than averaged across the year.

### 4.3. Prediction and Explanation Must Remain Separated

The operational-history model performed substantially better than weather-only or non-weight sensor-history models. This interpretation is consistent with earlier multimodal hive-weight forecasting, in which historical weight measurements were the dominant predictive inputs and environmental and acoustic telemetry provided complementary context [17]. That study addressed a continuous forecasting target; its regression metrics are not directly comparable with the event-discrimination metrics reported here. This motivates further evaluation of an alerting system, but is also expected: lagged weight-derived features preserve recent information from the same physical process used to define the target. The model is therefore an autoregressive operational forecaster. It should not be presented as an independent biological explanation of the processes producing short-term hive-mass increase.

Conversely, the lower performance of weather and non-weight sensors does not make them scientifically unimportant. Weather, temperature and acoustics may help identify why a prediction changes, distinguish plausible biological activity from technical artefacts, and support other endpoints such as colony-strength auditing. The two-layer interpretation prevents a common category error: ranking explanatory value solely by predictive importance.

The weaker non-weight models may also reflect measurement heterogeneity rather than weak biological relevance alone. Microphone position, hardware revision, firmware and FFT configuration were not recoverable; acoustic amplitude was uncalibrated and analysed in relative units. Likewise, 25.2% of days with a valid internal-humidity daily maximum had a maximum above 80% RH, and no device-specific drift correction was available. Training-fold imputation and standardisation harmonised numerical scale for modelling but cannot reconstruct physical calibration or remove placement-specific bias.

Fifty repeated stream-grouped splits showed that the reported discrimination was not an artefact of one favourable partition, although split-to-split variation was larger for average precision in autumn. The previous-day mass-change persistence score was already strong, as expected for an autoregressive target, but paired whole-stream bootstrap intervals supported an AP improvement in all four seasons and an AUC improvement in three. The autumn AUC-difference interval included zero. Unweighted histogram gradient boosting was retained as the primary model because balanced sample weights did not materially improve ROC AUC or average precision and generally worsened the Brier score. Thresholds selected using training data improved sensitivity in lower-prevalence periods, but these cutoffs and the internal reliability diagrams require external recalibration before deployment. Rounded-location-grouped sensitivity results were similar, yet true apiary-level independence remains unverified.

### 4.4. Implications for Intelligent Sensing Systems

For longitudinal sensing systems, channel interpretation and data provenance should be established before predictive outputs are used operationally. For a practical smart-hive system, the proposed sequence is as follows:

1. Verify channel behaviour and data sufficiency;
2. Infer a cautious sensor role;
3. Derive season-aware biological-context indicators;
4. Generate a short-horizon operational score from strictly lagged inputs;
5. Expose provenance and uncertainty so a beekeeper can distinguish a contextual alert from a direct measurement.

This structure is compatible with calls for predictive, decision-oriented monitoring while preserving the limitations of proxy labels [4,44].

The practical use of integrated hive-mass, microclimate and acoustic telemetry in apiary management and agricultural advisory services has also been discussed in a review and BeeHUB case study [57].

## 5. Limitations

The following limitations define the evidential scope. First, the operational target was derived from daytime mass change and was not validated against floral phenology, direct nectar intake, forager traffic or inspection labels. The 0.3 kg cutoff is pragmatic rather than a biological boundary, although sensitivity was examined across thresholds from 0.10 to 1.00 kg.

Second, hive_id is a device/telemetry-stream identifier, not a verified longitudinal colony identity. Stream-level grouping prevents the same recorded identifier from occurring in training and test data, but device transfer or replacement could allow one biological colony to be represented by different identifiers.

Third, sensor-role inference has behavioural plausibility but no technician-verified placement ground truth. Scores and mapping-confidence classes are heuristic rather than calibrated probabilities, and a time-invariant role can mix different placements or colony conditions across years. A mapping error propagates into inferred internal temperature, thermal buffer and state assignment.

Fourth, only 110 of 732 nonblank role-audit streams entered the biological table. The retained subset differed modestly from the 98 role-eligible low-confidence streams, so state frequencies and biological contrasts are conditional on data completeness and the mapping rule. Maintenance quality was unavailable and cannot be inferred.

Fifth, the calendar periods are not site-specific phenological labels. The weather/modelling subset covered 42.08–54.58° N and 0.49–24.74° E, with incomplete country and altitude metadata; conclusions about May–June should not be generalised to all of temperate Europe.

Sixth, abrupt scale discontinuities were suppressed or flagged, but feeding, harvesting, hive opening, equipment changes and gradual scale drift were not independently labelled. Outcome availability also differed among thermal states, particularly for unknown, ambient-like/empty and cold/low-activity days.

Seventh, repeated grouped validation, rounded-location sensitivity analyses and whole-stream bootstrap intervals improve internal robustness but do not replace external validation. Rounded coordinates are not verified apiary identifiers, and shared weather, management or forage can remain across stream-grouped partitions. Canonical-split bootstrap intervals are conditional on one partition, whereas split-to-split standard deviations address a different source of uncertainty.

Eighth, device-specific scale calibration, microphone calibration, verified sensor-position logs and longitudinal drift or maintenance records were not available. Microphone hardware and placement may vary, acoustic amplitude is relative, and internal-humidity measurements frequently exceeded the sensor’s normal 0–80% RH range. Training-fold imputation and standardisation do not correct these physical measurement uncertainties.

Finally, the corrected feature pipeline enforces an explicit midnight observation-time origin, but the stream cohort was selected retrospectively and the actual release times of historical weather products were unavailable. Past-only roles use each held-out stream’s own prior unlabelled measurements; these results do not represent cold-start performance without such history. Grouped partitions also span the same calendar years and do not test future-year distribution shifts. Accordingly, the results do not establish sensor-position diagnostic accuracy, a causal thermal mechanism, direct nectar-intake measurement, unseen-colony generalisability or deployment-ready probability calibration. External studies should combine verified sensor and colony histories, field-labelled events, apiary-level grouping and independent calibration data.

## 6. Conclusions

Multi-year smart-hive telemetry contains distinct semantic, contextual and predictive information. Behaviour-based temperature-channel interpretation can provide plausible context when archived channel names are unreliable, but the inferred roles remain heuristic, time-aggregated and unvalidated against physical placement. In the selected 110-stream subset, warm-stable days in the calendar-defined May–June period were associated with higher median daytime mass change and event rate than four common comparison states. This association did not extend to the sparse warm-unstable state and should not be interpreted as a biological mechanism.

Across 50 held-out-stream splits, the primary strictly lagged unweighted-HGB model achieved mean ROC AUCs of 0.748–0.809 and mean average precisions of 0.611–0.793. A simple previous-day persistence score was strong, confirming the autoregressive nature of the task, but the full model improved canonical-split ROC AUC by 0.049–0.084, with whole-stream AUC-difference intervals excluding zero in three periods; the autumn interval included zero. AP-difference intervals were positive in all four periods. These results support further external validation of semantic role inference and lagged operational forecasting. They do not establish direct nectar-intake measurement, causal thermal effects, colony-health diagnosis, unseen-colony generalisability or deployment-ready calibration.

## Figures and Tables

**Table 1:** Seasonal sizes of the final biological and strict temporal modelling tables. Modelling rows required a non-missing cleaned target and weather match.

Season	Months	Biological Days	Streams	Weather Days	Model Days	Events	Non-Events	Model Streams
Early spring	March–April	6381	103	5118	3506	1117	2389	76
May–June period	May–June	8825	110	6956	5083	2431	2652	81
Summer	July–August	10,041	110	7917	5828	2086	3742	83
Autumn	September–October	9757	110	7765	5617	1458	4159	83
Total	–	35,004	110 *^a^*	27,756	20,034	7092	12,942	83 *^a^*

*^a^* Maximum within-period number of unique nonblank telemetry-stream identifiers. These entries report the largest count among the four calendar periods, not the number of distinct identifiers across their union. The period-specific sets overlap.

**Table 2:** BeeHUB sensing modalities represented in the analytical archive. Hardware information is reported only where supported by retained documentation; NR denotes information not retained in the frozen export.

Modality	Archive Representation	Interpretation and Metrological Status
Hive mass	Absolute weight and derived within-day or between-day changes	Custom 45 cm × 45 cm frame with 50 kg strain-gauge load cells. Supplier rating per sensing element: 0.2% FS comprehensive error, 1.1±0.15 mV/V sensitivity, −10 to +50 °C operating range, and 150% FS overload. System-level calibration and drift records: NR.
Temperature	DS18B20 probes; ambient and nominal internal channels, including temp_brood_c and temp_inside2_c	1-Wire digital thermometer; −55 to +125 °C range, ±0.5 °C accuracy from −10 to +85 °C, and 9–12-bit programmable resolution [49]. Placement audit: NR; channel role was therefore inferred from behaviour.
Relative humidity	SHT20 internal relative-humidity channel (%)	I^2^C sensor; 0–100% RH extended range, typical ±3.0% RH accuracy at 25 °C, and 0.04% RH resolution at 12 bits [50]. Device-specific calibration records: NR; used as contextual telemetry only.
Acoustics	Aggregated frequency and amplitude indicators	Feature-level representation of colony sound, not raw audio or calibrated sound-pressure level. The documented BeeHUB design uses an I^2^S MEMS microphone and on-node spectral processing; row-level hardware revision and amplitude calibration: NR.

**Table 3:** Flow of source files, telemetry-stream identifiers and stream-days through the analysis. Channel availability denotes at least one non-missing observation before biological range filtering; streams may contribute to multiple modalities.

Stage or Archive Channel	Files or Streams	Rows or Stream-Days
*Archive construction*		
Source files detected by the broad discovery scan	1286 files	–
Successfully loaded telemetry source files	1241 files	20,963,690 raw rows
Empty or near-empty telemetry files	40 files	0 loaded rows
Out-of-scope cached weather files	5 files	–
Standardised stream groups	1032 *^a^*	–
Deduplicated hourly archive	1032 *^a^*	7,314,640 stream-hours
*Channel and stream availability*		
Weight channel present before biological filtering	959	–
Streams eligible for weight analysis	736	–
temp_brood_c present before biological filtering	1029	–
temp_inside2_c present before biological filtering	756	–
temp_out_c present before biological filtering	263	–
Internal humidity present before biological filtering	1004	–
Acoustic frequency/amplitude present before biological filtering	979/911	–
*Temperature-channel role inference*		
Nonblank streams entering the temperature-role audit	732	–
Raw role-audit evaluations, including two blank-ID rows	1466 evaluations	–
Streams with at least 30 valid joint channel–ambient days	208	–
High- or medium-confidence internal-channel mapping	110	–
Eligible but low-confidence mappings	98	–
*Final analytical datasets*		
Final active-season biological table	110	35,004 stream-days
Weather-matched biological table	110	27,756 stream-days
Strict temporal modelling table	83 (maximum/season)	20,034 stream-days

*^a^* Includes 1031 nonblank identifiers and one blank-identifier group; the latter was excluded from all downstream revised analyses.

**Table 4:** Exact behaviour-based sensor-role decision rule. The first satisfied row was assigned.

Assigned Role	Exact Condition	Minimum Days
Insufficient data	N<30	<30
Ambient-like	$S_{\mathrm{ext}}\geq 0.65$, $S_{\mathrm{ext}}\geq S_{\mathrm{int}}$ and $S_{\mathrm{ext}}\geq S_{\mathrm{core}}$	30
Colony-core-like	Previous rule false, $S_{\mathrm{core}}\geq 0.55$ and $S_{\mathrm{core}}\geq S_{\mathrm{int}}$	30
Internal-hive-like	Previous rules false and $S_{\mathrm{int}}\geq 0.45$	30
Mixed or uncertain	None of the preceding rules	30

**Table 5:** Exact ordered rules for daily thermal-state assignment. $T_{i}$ is inferred internal mean temperature, *B* is the thermal buffer and $s_{i}$ is the daily standard deviation of hourly inferred internal temperature.

Order	State	First-Match Condition
1	Unknown	$T_{i}$, ambient mean, *B* or $s_{i}$ is missing
2	Cold or low activity	$T_{i}<12\;{}^{\circ}C$
3	Ambient-like or empty	|B|<3 °C and $T_{i}<25\;{}^{\circ}C$
4	Active internal	$20\leq T_{i}<30\;{}^{\circ}C$ and B≥5 °C
5	Warm unstable	$T_{i}\geq 28\;{}^{\circ}C$, $s_{i}$ observed and $s_{i}>5\;{}^{\circ}C$
6	Warm stable	$T_{i}\geq 32\;{}^{\circ}C$, B≥8 °C, and $s_{i}\leq 5\;{}^{\circ}C$
7	Mixed or uncertain	None of the preceding conditions

**Table 6:** Canonical seed-42 held-out-stream split used for exact test counts, calibration, whole-stream uncertainty intervals and paired model comparisons. The primary performance estimates are means and standard deviations across 50 repeated grouped splits.

Season	Train Days	Test Days	Train Streams	Test Streams	Train Event Rate	Test Event Rate
Early spring	2607	899	57	19	0.343	0.248
May–June	3765	1318	60	21	0.487	0.454
Summer	4298	1530	62	21	0.372	0.317
Autumn	4068	1549	62	21	0.266	0.243

**Table 7:** All inferred thermal states in the calendar-defined May–June period. Event rates and median daytime mass changes use only days with an observed daytime mass change; stream counts therefore refer to streams with at least one gain-valid day in the corresponding state.

State	All State-Days	Gain-Valid Streams	Gain-Valid Days	Median Change (kg)	Event Rate (%)	Median $T_{i}$/Buffer (°C)
Warm stable	5070	96	4372	0.350	52.5	34.8/17.0
Active internal	791	66	641	0.150	34.9	27.5/10.3
Mixed or uncertain	1179	78	906	0.150	41.4	30.9/8.6
Ambient-like or empty	830	30	330	0.000	20.9	20.5/0.2
Cold or low activity	253	34	102	0.000	18.6	8.9/3.1
Warm unstable	64	24	48	0.525	54.2	32.0/17.0
Unknown	638	18	158	0.400	52.5	27.7/10.2

**Table 8:** May–June warm-stable contrasts from 2000 whole-stream bootstrap resamples. The stream columns show the numbers with gain-valid observations in the warm-stable and comparison states; event-rate differences are in percentage points.

Comparison State	Streams (Warm/Comparison)	Median-Change Difference, kg (95% CI)	Event-Rate Difference, pp (95% CI)
Active internal	96/66	0.200 (0.100, 0.350)	17.5 (10.2, 25.3)
Mixed or uncertain	96/78	0.200 (0.075, 0.300)	11.1 (4.2, 18.7)
Ambient-like or empty	96/30	0.350 (0.250, 0.500)	31.6 (19.7, 43.4)
Cold or low activity	96/34	0.350 (0.225, 0.500)	33.8 (22.9, 43.7)
Warm unstable	96/24	−0.175 (−0.825, 0.150)	−1.7 (−16.6, 14.3)

**Table 9:** Primary unweighted-HGB all-history results. Counts and bracketed whole-stream 95% confidence intervals refer to the canonical seed-42 split; means and standard deviations refer to 50 repeated grouped splits. AP denotes average precision and BS the Brier score. Probability cutoffs and operating metrics are reported in Supplementary Table S12.

Season	Days	Events	Streams	ROC AUC Mean ± SD	Canonical AUC [95% CI]	AP Mean ± SD	Canonical AP [95% CI]	BS Mean ± SD
Early spring	899	223	19	0.748 ± 0.020	0.728 [0.684, 0.768]	0.624 ± 0.044	0.533 [0.453, 0.609]	0.189 ± 0.018
May–June	1318	598	21	0.809 ± 0.018	0.800 [0.751, 0.841]	0.793 ± 0.032	0.758 [0.692, 0.808]	0.181 ± 0.010
Summer	1530	485	21	0.797 ± 0.026	0.826 [0.752, 0.876]	0.712 ± 0.056	0.729 [0.560, 0.831]	0.169 ± 0.016
Autumn	1549	376	21	0.783 ± 0.031	0.779 [0.677, 0.828]	0.611 ± 0.091	0.601 [0.313, 0.729]	0.147 ± 0.017

**Table 10:** Repeated grouped performance of the full unweighted-HGB model and the previous-day mass-change persistence baseline. Full/persistence columns show means ± SD across 50 splits. Paired differences and 95% whole-stream bootstrap intervals refer to the canonical split.

Season	Full AUC	Persistence AUC	ΔAUC [95% CI]	Full AP	Persistence AP	ΔAP [95% CI]
Early spring	0.748 ± 0.020	0.681 ± 0.017	0.084 [0.042, 0.118]	0.624 ± 0.044	0.524 ± 0.050	0.128 [0.077, 0.174]
May–June	0.809 ± 0.018	0.751 ± 0.018	0.077 [0.050, 0.104]	0.793 ± 0.032	0.711 ± 0.035	0.087 [0.048, 0.128]
Summer	0.797 ± 0.026	0.727 ± 0.030	0.060 [0.026, 0.088]	0.712 ± 0.056	0.581 ± 0.066	0.127 [0.066, 0.180]
Autumn	0.783 ± 0.031	0.708 ± 0.033	0.049 [−0.003, 0.095]	0.611 ± 0.091	0.463 ± 0.085	0.108 [0.028, 0.153]

## Data Availability

The de-identified daily data supporting the findings are available from the corresponding author upon reasonable request. Raw device telemetry and precise apiary coordinates are not publicly available because they contain commercially sensitive information and confidential location data governed by platform-user agreements.

## Supplementary Materials

The following supporting information can be downloaded at: https://www.mdpi.com/article/10.3390/s26216684/s1, Table S1, Archive, identifier, and mass-unit audit. The blank identifier group was included only in the archive audit and excluded from all revised biological and predictive analyses; Table S2, Geographic scope and rounded-location-grouped predictive sensitivity. Coordinates were rounded and are not verified apiary identifiers. AUC and AP are means across 50 grouped splits of the primary unweighted-HGB model; Table S3, Role-parameter provenance and perturbation sensitivity. Values were fixed in the rule-based pipeline; no labelled placement set and no outcome tuning were used; Table S4, Role-score uncertainty, annual stability, and restricted-mapping May–June sensitivity. Differences compare warm-stable with the stated comparison state; intervals are from 2000 whole-stream bootstrap draws; Table S5, Selection audit for the role-corrected biological subset. Event rates use the 0.3-kg operational threshold. Groups share March–October within 4 January 2022–2 December 2025 and the accepted gain range −5 to 10 kg. Stream counts denote contributors with valid mass data; Table S6, All May–June thermal states and rule-order sensitivity. Outcome summaries use gain-valid days; Table S7, Summary of biological robustness analyses for the May–June warm-stable contrast; Table S8, Exact same-day contextual model results on the canonical held-out-stream split. Each value is the better of the two prespecified estimators within the feature family and is therefore descriptive; Table S9, Corrected grouped predictive validation. Values are means ± SD across 50 splits. Canonical-split whole-stream intervals are reported separately in the main predictive table and Data S1; Table S10, Acoustic-amplitude and internal-relative-humidity preprocessing audit within 4 January 2022–2 December 2025. Amplitude summaries use daily medians; humidity exposure uses daily maxima; Table S11, Corrected lagged feature-family comparisons with the same unweighted HGB specification and the same 50 grouped partitions. Values are means ± SD. All rows retain identical target observations within a split; Table S12, Operating metrics at the fixed probability cutoff 0.5 and a cutoff selected entirely within each outer training partition. Values are means across the same 50 stream-grouped partitions. BA denotes balanced accuracy; Figure S1, Reliability diagrams for the operational classifiers on the canonical held-out-stream splits. Eight quantile-based bins were used. These are internal diagnostics; no post-hoc probability calibrator was fitted; Figure S2, Sensitivity of predictive performance to the operational daytime mass-increase threshold from 0.10 to 1.00 kg. Results use 20 repeated grouped splits per period for the primary unweighted-HGB model and lag-1 persistence baseline; Data S1, machine-readable revision outputs.

## Abbreviations

The following abbreviations are used in this manuscript:

AIoT	Artificial Intelligence of Things
AP	Average precision
BS	Brier score
CI	Confidence interval
F1	F1 score: harmonic mean of precision and recall
FFT	Fast Fourier transform
FS	Full scale
HGB	Histogram gradient boosting
I^2^C	Inter-integrated circuit
I^2^S	Inter-IC Sound
ID	Identifier
IoT	Internet of Things
LTE-M	Long-Term Evolution for Machines
MEMS	Microelectromechanical systems
NB-IoT	Narrowband Internet of Things
NR	Not retained in the frozen export
pp	Percentage points
RH	Relative humidity
ROC AUC	Area under the receiver operating characteristic curve
SD	Standard deviation
UTC	Coordinated Universal Time
