# Supplementary material for: Behaviour-Based Temperature-Sensor Role Inference and Lagged Weight-Gain Event Prediction in Multi-Year Smart-Hive Telemetry
Source: Sensors (Basel). 2026 Oct 22;26(21):6684. doi: 10.3390/s26216684 (PMC13765559; doi:10.3390/s26216684)
Supplement: Supplementary file 1 [file sensors-26-06684-s001.zip › Supplementary Material_MDPI_Sensors.pdf]

Supplementary Material
Behaviour-Based Temperature-Sensor Role Inference and Lagged Weight-Gain Event Prediction in Multi-Year Smart-Hive Telemetry

Sebastian Gorecki, Sławomir Hausman, and Piotr Korbel

Revision: October 2026

This revision supplement reports the additional audits and sensitivity analyses requested during peer review. The archive field `hive_id` is treated as a device/telemetry-stream identifier rather than a verified longitudinal colony identity. All references to streams and stream-days follow that operational definition. Archive-wide counts are distinguished from the 2022–2025 biological and predictive interval. Predictive results below use the corrected midnight observation-time feature pipeline; same-day contextual results retain their separate retrospective interpretation.

Table S1: Archive, identifier, and mass-unit audit. The blank identifier group was included only in the archive audit and excluded from all revised biological and predictive analyses.

**Audit item**	**Count**
Source files detected / telemetry files loaded	1,286 / 1,241
Empty or near-empty telemetry files / out-of-scope weather caches	40 / 5
Standardised groups: nonblank / blank	1,031 / 1
Raw rows / deduplicated stream-hours	20,963,690 / 7,314,640
Nonblank streams in role audit / eligible role streams / retained streams	732 / 208 / 110
Blank-ID role rows / blank-ID active-season rows removed	2 / 328
Corrected biological / weather-matched / modelling stream-days	35,004 / 27,756 / 20,034
Raw median 5–250 (kg assumed) / 5,000–250,000 (divided by 1,000)	723 / 52
Raw median below 5 / 250–5,000 / above 250,000 / missing	178 / 6 / 0 / 73
Weight-eligible streams after all criteria: kg-range / converted range	692 / 44

Table S2: Geographic scope and rounded-location-grouped predictive sensitivity. Coordinates were rounded and are not verified apiary identifiers. AUC and AP are means across 50 grouped splits of the primary unweighted-HGB model.

**Scope or period**	**Streams**	**Rounded locations**	**AUC**	**AP**
Entire weather/modelling subset	87	80	–	–
Latitude < 46°N	2	2	–	–
Latitude 46–< 50°N	21	19	–	–
Latitude 50–< 55°N	64	59	–	–
Early spring	19.06[a]	18.0[a]	0.749	0.616
May–June	20.42[a]	19.0[a]	0.804	0.787
Summer	20.62[a]	19.0[a]	0.798	0.710
Autumn	20.62[a]	19.0[a]	0.784	0.606

Coordinate span: 42.08–54.58°N and 0.49–24.74°E. [a] Mean held-out count across 50 splits.

Table S3: Role-parameter provenance and perturbation sensitivity. Values were fixed in the rule-based pipeline; no labelled placement set and no outcome tuning were used.

**Component**	**Published values**	**Interpretive rationale**
S_{ext} weights	0.45 correlation; 0.35 ambient offset; 0.20 range ratio	Emphasise ambient co-movement
S_{int} weights	0.30 warm offset; 0.30 plausible temperature; 0.20 decorrelation; 0.20 range	Balanced internal-placement evidence
S_{core} weights	0.35 core temperature; 0.25 stability; 0.25 warm offset; 0.15 decorrelation	Warm, stable core-like behaviour
Temperature supports	Internal 12–38 °C, peak 30 °C; core 26–37.5 °C, peak 34.5 °C	Broad internal and narrower brood/core plausibility
Role thresholds	External 0.65; core 0.55; internal 0.45; high-confidence internal score 0.60	Conservative first-match hierarchy
Perturbation results	Eligible-role agreement 93.1–100%; selected-sensor agreement 99.5–100%; 103–114 retained streams	Cutoffs ±0.05, scales/ranges ±10%, peaks ±1 °C, and equal weights

Table S4: Role-score uncertainty, annual stability, and restricted-mapping May–June sensitivity. Differences compare warm-stable with the stated comparison state; intervals are from 2,000 whole-stream bootstrap draws.

**Mapping subset**	**Comparison state**	**Mapping streams**	**Median-change difference, kg**	**95% CI**	**Event-rate difference, pp**
All retained	Active internal	110	0.200	0.100, 0.350	17.5
All retained	Mixed or uncertain	110	0.200	0.075, 0.300	11.1
All retained	Ambient-like or empty	110	0.350	0.250, 0.500	31.6
High confidence	Active internal	85	0.250	0.100, 0.400	19.6
High confidence	Mixed or uncertain	85	0.250	0.100, 0.350	11.8
High confidence	Ambient-like or empty	85	0.400	0.275, 0.650	36.7
Score margin ≥ 0.10	Active internal	83	0.175	0.075, 0.300	14.8
Score margin ≥ 0.10	Mixed or uncertain	83	0.175	0.050, 0.300	10.7
Score margin ≥ 0.10	Ambient-like or empty	83	0.325	0.200, 0.450	35.6
Stable multiyear	Active internal	26	0.175	−0.000, 0.450	15.0
Stable multiyear	Mixed or uncertain	26	0.125	−0.125, 0.313	5.5
Stable multiyear	Ambient-like or empty	26	0.325	0.050, 0.681	42.3
High and stable	Active internal	20	0.250	0.000, 0.600	18.1
High and stable	Mixed or uncertain	20	0.200	−0.050, 0.450	9.3
High and stable	Ambient-like or empty	20	0.450	0.100, 0.879	47.3

Stream counts in this table describe mapping-subset rosters; gain-valid stream counts vary by comparison. Among 216 eligible channel evaluations, 38 (17.6%) had a top-versus-second score margin below 0.10. Among 110 selected streams, 27 (24.5%) had a selected-sensor margin below 0.10. Of 67 selected mappings evaluable in at least two years, 41 (61.2%) changed annual behaviour-based role.

Table S5: Selection audit for the role-corrected biological subset. Event rates use the 0.3-kg operational threshold. Groups share March–October within 4 January 2022–2 December 2025 and the accepted gain range −5 to 10 kg. Stream counts denote contributors with valid mass data.

**Group**	**Streams**	**Gain-valid days**	**Median change / event rate**
Retained high/medium confidence	110	28,544	0.100 kg / 33.9%
Eligible low confidence	98	20,790	0.050 kg / 28.4%
All nonretained with usable mass	514	130,659	0.100 kg / 33.5%

Retained minus eligible-low: median difference 0.050 kg (95% CI −0.050, 0.100); event-rate difference 5.48 pp (0.80, 10.28). Retained minus all nonretained: median difference 0.000 kg (−0.050, 0.035); event-rate difference 0.36 pp (−3.68, 4.42). The all-nonretained roster contained 622 streams; 514 contributed valid days in this comparison window.

Table S6: All May–June thermal states and rule-order sensitivity. Outcome summaries use gain-valid days.

State	All days	Gain-valid streams	Gain-valid days	Median change, kg	Event rate, %	Median T_i/buffer, °C
Warm stable	5,070	96	4,372	0.350	52.5	34.8 / 17.0
Active internal	791	66	641	0.150	34.9	27.5 / 10.3
Mixed or uncertain	1,179	78	906	0.150	41.4	30.9 / 8.6
Ambient-like or empty	830	30	330	0.000	20.9	20.5 / 0.2
Cold or low activity	253	34	102	0.000	18.6	8.9 / 3.1
Warm unstable	64	24	48	0.525	54.2	32.0 / 17.0
Unknown	638	18	158	0.400	52.5	27.7 / 10.2

Reversing the active-internal and warm-unstable rule order changed 94 of 35,004 stream-days (0.27%), including 28 of 8,825 May–June days (0.32%); all changed records moved from active internal to warm unstable.

Table S7: Summary of biological robustness analyses for the May–June warm-stable contrast.

Diagnostic	Result
Ambient-temperature stratification	Warm-stable median-change differences: 0.413 kg vs ambient-like/empty; 0.143 kg vs mixed/uncertain; 0.195 kg vs active internal.
Within-stream comparison	Median differences: 0.250, 0.237, and 0.250 kg; positive in 19/23, 50/69, and 42/60 paired streams, respectively.
Year-specific summaries	Median contrasts remained positive in all available years for the three principal comparisons.
Minimum stream-day and top-contributor restrictions	Direction persisted for thresholds of 10–90 days and after excluding the top 1%, 2%, 5%, or 10% of contributing streams.
Random stream subsampling	Positive in all 300 runs per principal comparison across fractions 50%, 65%, and 80%.
Operational threshold 0.10–1.00 kg	Event-rate difference positive at all ten thresholds for active internal (10.0–18.3 pp), mixed/uncertain (7.9–11.1 pp), ambient-like/empty (16.2–32.4 pp), and cold/low activity (21.5–33.8 pp); negative and imprecise for warm unstable (−18.4 to −1.7 pp).

Table S8: Exact same-day contextual model results on the canonical held-out-stream split. Each value is the better of the two prespecified estimators within the feature family and is therefore descriptive.

**Period**	**Weather only**		**Non-weight BeeHUB**		**Combined**	
	**AUC**	**AP**	**AUC**	**AP**	**AUC**	**AP**
Early spring	0.700	0.463	0.661	0.444	0.716	0.480
May–June	0.648	0.576	0.665	0.601	0.694	0.627
Summer	0.575	0.372	0.565	0.351	0.562	0.369
Autumn	0.521	0.255	0.532	0.254	0.500	0.235

Current-day and future mass-change features were excluded. The weather-only representations comprised 32 current-day numeric variables or 104 current, lagged, rolling, and calendar variables; non-weight BeeHUB comprised 16 numeric and one categorical variable; the combined representation comprised 118 numeric and one categorical variable. Complete estimator-specific results and exact same-day feature lists are supplied in Data S1; the table above is a compact summary, not the complete estimator comparison.

Table S9: Corrected grouped predictive validation. Values are means ± SD across 50 splits. Canonical-split whole-stream intervals are reported separately in the main predictive table and Data S1.

**Period**	**Model**	**ROC AUC**	**AP**	**Brier score**	**Mean selected cutoff**
Early spring	HGB, unweighted	0.748±0.020	0.624±0.044	0.189±0.018	0.217
Early spring	HGB, balanced weights	0.746±0.019	0.622±0.043	0.189±0.016	0.266
Early spring	Logistic, balanced	0.702±0.021	0.565±0.047	0.220±0.012	0.524
Early spring	Lag-1 persistence	0.681±0.017	0.524±0.050	–	0.300 kg
May–June	HGB, unweighted	0.809±0.018	0.793±0.032	0.181±0.010	0.473
May–June	HGB, balanced weights	0.809±0.018	0.793±0.033	0.181±0.011	0.498
May–June	Logistic, balanced	0.773±0.024	0.753±0.036	0.195±0.012	0.497
May–June	Lag-1 persistence	0.751±0.018	0.711±0.035	–	0.300 kg
Summer	HGB, unweighted	0.797±0.026	0.712±0.056	0.169±0.016	0.311
Summer	HGB, balanced weights	0.796±0.027	0.712±0.057	0.171±0.016	0.386
Summer	Logistic, balanced	0.765±0.029	0.652±0.065	0.194±0.015	0.468
Summer	Lag-1 persistence	0.727±0.030	0.581±0.066	–	0.300 kg
Autumn	HGB, unweighted	0.783±0.031	0.611±0.091	0.147±0.017	0.180
Autumn	HGB, balanced weights	0.780±0.032	0.607±0.092	0.150±0.015	0.274
Autumn	Logistic, balanced	0.764±0.028	0.547±0.084	0.188±0.015	0.496
Autumn	Lag-1 persistence	0.708±0.033	0.463±0.085	–	0.300 kg

Canonical paired HGB-minus-persistence AUC differences were 0.084 [0.042, 0.118], 0.077 [0.050, 0.104], 0.060 [0.026, 0.088], 0.049 [-0.003, 0.095] from early spring through autumn. Complete split-level metrics, paired bootstrap draws, complete-case comparisons and canonical uncertainty intervals for every classifier and feature family are in Data S1. No post-hoc probability calibrator was fitted.

Table S10: Acoustic-amplitude and internal-relative-humidity preprocessing audit within 4 January 2022–2 December 2025. Amplitude summaries use daily medians; humidity exposure uses daily maxima.

**Modality**	**Streams**	**Sensor-years**	**Daily records**	**Additional audit**
Relative acoustic amplitude	601	1,240	190,835	Median 19 a.u.; no physical calibration or drift correction
Internal RH daily maximum	632	1,299	200,195	Median 63.3% RH; 25.2% of days had maximum > 80% RH; no drift correction

Table S11: Corrected lagged feature-family comparisons with the same unweighted HGB specification and the same 50 grouped partitions. Values are means ± SD. All rows retain identical target observations within a split.

**Period**	**Feature family**	**ROC AUC**	**AP**
Early spring	Weather history	0.636±0.030	0.485±0.045
Early spring	Non-weight sensor history	0.599±0.029	0.432±0.057
Early spring	Weight history	0.745±0.024	0.605±0.048
Early spring	All history	0.748±0.020	0.624±0.044
May–June	Weather history	0.619±0.030	0.584±0.061
May–June	Non-weight sensor history	0.569±0.027	0.541±0.060
May–June	Weight history	0.783±0.020	0.749±0.033
May–June	All history	0.809±0.018	0.793±0.032
Summer	Weather history	0.569±0.027	0.433±0.059
Summer	Non-weight sensor history	0.511±0.033	0.378±0.064
Summer	Weight history	0.792±0.029	0.698±0.059
Summer	All history	0.797±0.026	0.712±0.056
Autumn	Weather history	0.542±0.032	0.298±0.063
Autumn	Non-weight sensor history	0.558±0.043	0.325±0.084
Autumn	Weight history	0.785±0.030	0.596±0.088
Autumn	All history	0.783±0.031	0.611±0.091

Numeric/categorical input counts including the two shared calendar terms: weather 118/0; non-weight sensors 58/4; weight 18/0; all history 190/4. An explicit midnight origin, exact date joins, dependency-aware mass-feature availability and past-only role mappings were used. The cohort remains retrospectively selected and historical weather release times were not verified.

Table S12: Operating metrics at the fixed probability cutoff 0.5 and a cutoff selected entirely within each outer training partition. Values are means across the same 50 stream-grouped partitions. BA denotes balanced accuracy.

**Period**	**Model**	**Selected cutoff**	**Sensitivity at 0.5**	**Specificity at 0.5**	**Sensitivity selected**	**Specificity selected**	**BA at 0.5**	**BA selected**
Early spring	HGB unweighted	0.217	0.406	0.897	0.667	0.680	0.652	0.673
Early spring	HGB balanced	0.266	0.485	0.848	0.687	0.654	0.667	0.671
Early spring	Logistic balanced	0.524	0.611	0.687	0.582	0.707	0.649	0.644
May–June	HGB unweighted	0.473	0.708	0.762	0.722	0.731	0.735	0.727
May–June	HGB balanced	0.498	0.715	0.753	0.711	0.745	0.734	0.728
May–June	Logistic balanced	0.497	0.686	0.720	0.686	0.707	0.703	0.697
Summer	HGB unweighted	0.311	0.559	0.871	0.703	0.742	0.715	0.723
Summer	HGB balanced	0.386	0.621	0.829	0.701	0.742	0.725	0.722
Summer	Logistic balanced	0.468	0.676	0.725	0.711	0.679	0.701	0.695
Autumn	HGB unweighted	0.180	0.393	0.941	0.669	0.740	0.667	0.705
Autumn	HGB balanced	0.274	0.490	0.892	0.669	0.731	0.691	0.700
Autumn	Logistic balanced	0.496	0.650	0.751	0.653	0.741	0.700	0.697

The selected cutoff maximised balanced accuracy in an inner stream-grouped validation subset. Imputation, scaling and encoding were fitted on the inner training subset, then refitted on all outer-training data with the classifier. No test outcomes were used to choose the cutoff. Cutoffs are internally estimated and are not universal deployment recommendations.

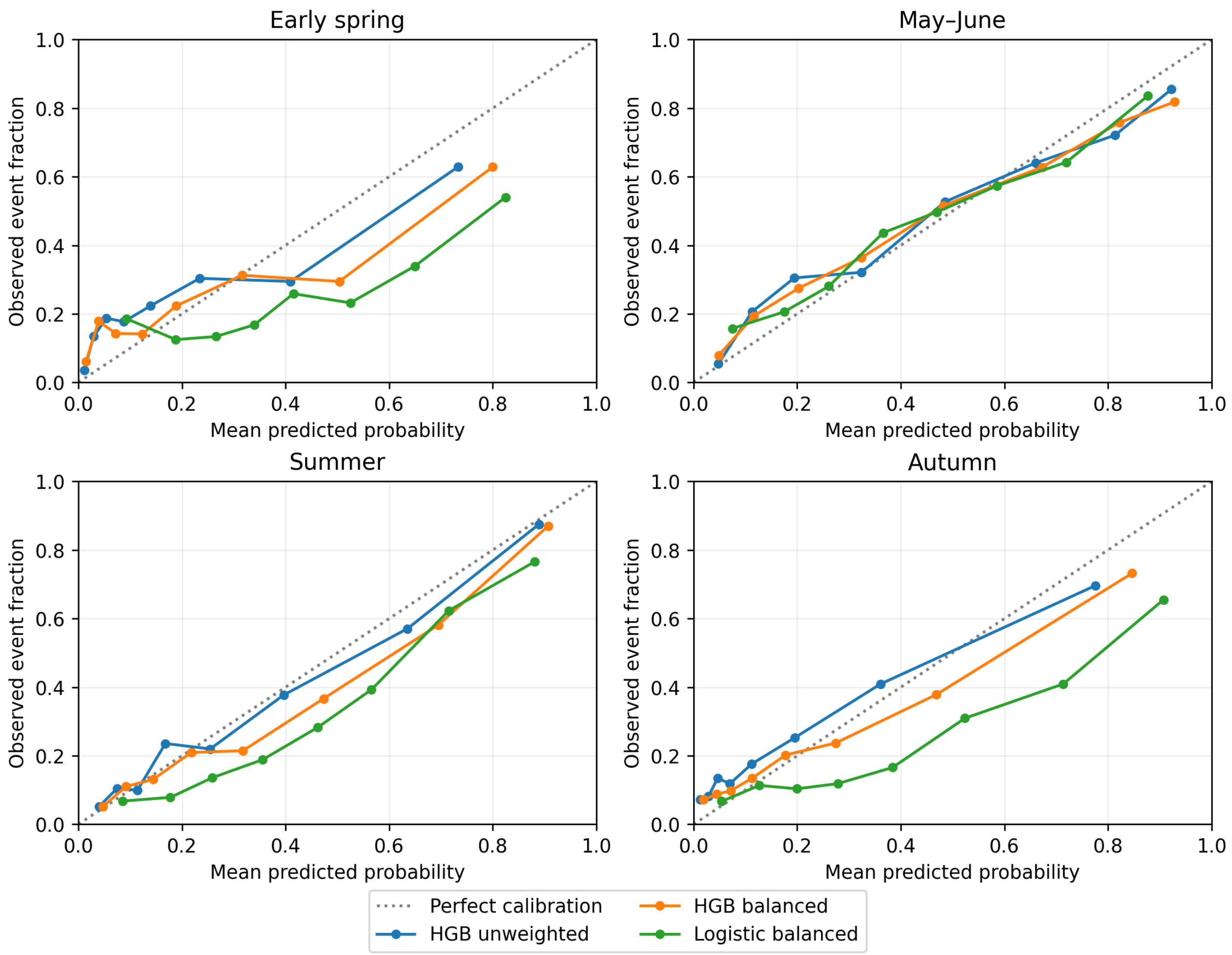

Figure S1: Reliability diagrams for the operational classifiers on the canonical held-out-stream splits. Eight quantile-based bins were used. These are internal diagnostics; no post-hoc probability calibrator was fitted.

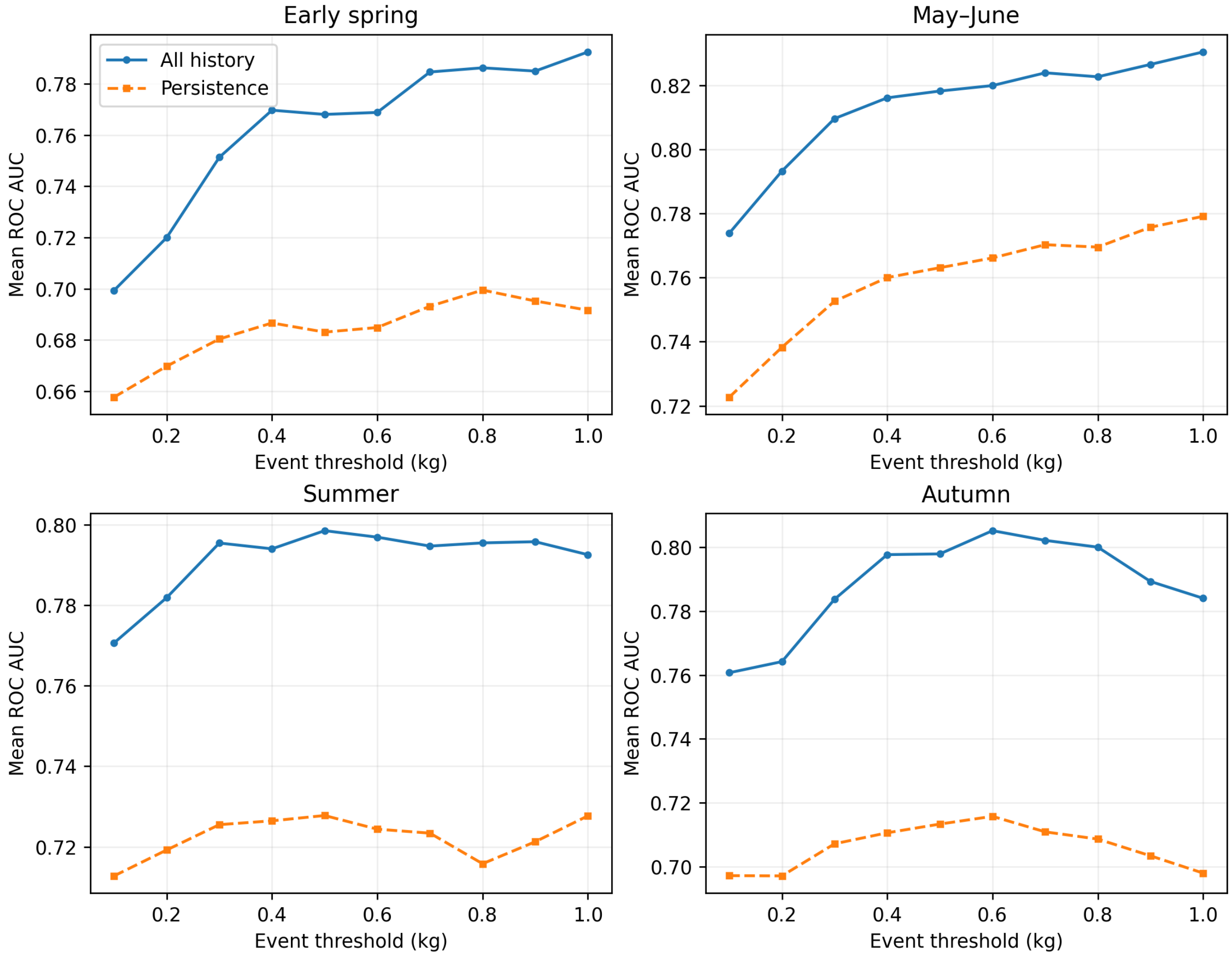

Figure S2: Sensitivity of predictive performance to the operational daytime mass-increase threshold from 0.10 to 1.00 kg. Results use 20 repeated grouped splits per period for the primary unweighted-HGB model and lag-1 persistence baseline.

Data S1: machine-readable revision outputs

Data S1 is supplied as `Data_S1_PRIVACY_SCREENED.zip`. It contains machine-readable analytical results, feature and software manifests, split-integrity audits, bootstrap results, calibration points, same-day model results, role and selection audits, and analysis code. The files and checksums of this shared version are listed in `FINAL_OUTPUT_MANIFEST.csv`.

Stream identifiers, location-group labels and identifying source filenames have been replaced with consistent, randomly assigned study-specific pseudonyms. Original identifiers, individual location coordinates, identifying source paths and a linkage key are not included. Selected detailed telemetry excerpts and date fields unnecessary for checking the reported results have been withheld; dates needed for temporal audits and some per-stream derived summaries remain. Numerical analytical estimates, prediction scores, target labels and saved train–test group memberships are unchanged. No models were retrained and no partitions were redrawn during preparation of this shared copy.

The disclosure scope and per-file changes are documented in `PRIVACY_RELEASE_NOTES.md` and `PRIVACY_VALIDATION_SUMMARY.json`. This is a pseudonymised release, not a claim of irreversible anonymisation. The code requires authorised access to the original inputs for full re-execution; those inputs remain subject to the restrictions stated in the manuscript's Data Availability Statement.
